# A multilevel statistical toolkit to study animal social networks: the Animal Network Toolkit Software (ANTs) R package

**DOI:** 10.1038/s41598-020-69265-8

**Published:** 2020-07-27

**Authors:** Sebastian Sosa, Ivan Puga-Gonzalez, Fenghe Hu, Jérôme Pansanel, Xiaohua Xie, Cédric Sueur

**Affiliations:** 1 Primate and Evolution Anthropology Laboratory, Anthropology Department, Sun Yat-Sen University, Guangzhou, China; 2 Université de Strasbourg, CNRS, IPHC UMR 7178, 67000 Strasbourg, France; 3 Institute for Religion, Philosophy and History, University of Agder, Kristiansand, Norway; 4 School of Data and Computer Science, Sun Yat-Sen University, Guangzhou, China; 5 Institut Universitaire de France, Paris, France

**Keywords:** Ecology, Zoology

## Abstract

The possible role played by individual attributes, sociodemographic characteristics and/or ecological pressures in the interaction between animals and the development of social relationships between them is of great interest in animal ecology and evolutionary biology. Social Network Analysis is an ideal tool to study these types of questions. The Animal Network Toolkit Software (ANTs) R package was specifically developed to provide all the different social network analysis techniques currently used in the study of animal social networks. This global package enables users to (1) compute global, polyadic and nodal network measures; (2) perform data randomisation: data stream and network (node and link) permutations; (3) perform statistical permutation tests for static or temporal network analyses, and (4) visualise networks. ANTs allows researchers to perform multilevel network analyses ranging from individual network measures to interaction patterns and the analysis of the overall network structure, and carry out static or temporal network analyses without switching between different R packages, thus making a substantial contribution to advances in the study of animal behaviour. ANTs outperforms existing R packages for the computation speed of network measures and permutations.

## Introduction

**Figure 1:**
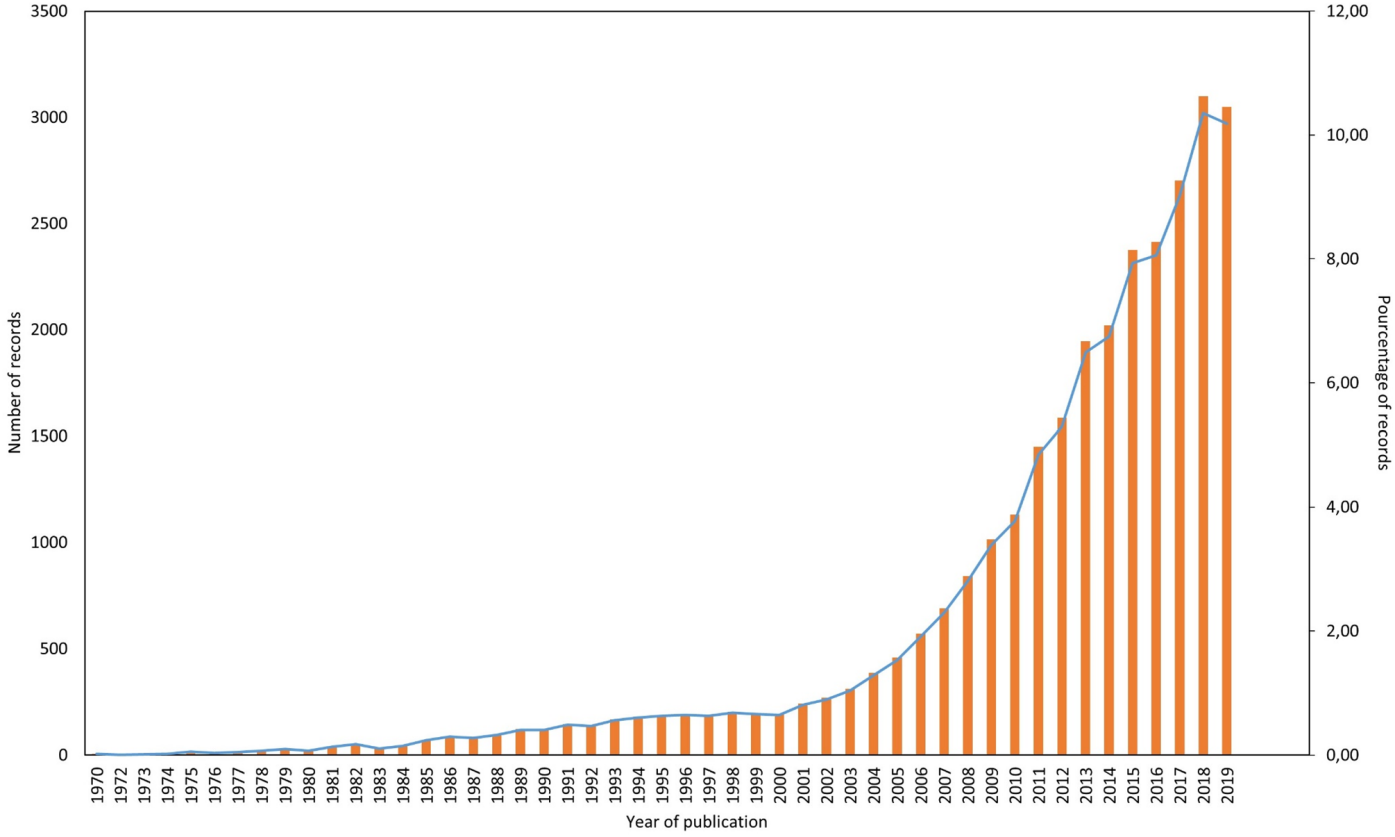
Record count and percentage of records of the keywords “social networks animals” per year. Source: Web of knowledge.

In recent years the use of network theory has developed considerably in animal research (Fig. 1), facilitating the study of relationships between individuals and in a variety of research fields, including genetic^1^, spatial movements^2^, animal groups and species assemblages^3^, epidemiology ^4^, information transmission ^5^, ethology, sociobiology^6^, evolution^7^, conservation^8^ and animal management^9^. Animal social networks are also dynamic as they change through time based on group composition and social choices/assemblages of individuals^10–13^. In addition, network properties may, as feedback, influence individuals’ health or fitness through social transmission of disease and/or information (diffusion processes) leading to changes in social relationships and, consequently, to changes in the network topology^14^. SNA allows the study of all these aspects and has revolutionised our understanding of complex intra- and interspecific interactions between individuals. However, while methods have been specifically developed, none of the current software allows a unique environment to handle animal social networks analysis.

A social network is composed of nodes (e.g. individuals, groups, populations or species) and edges that represent spatial associations or any type of social relationships (i.e. the total number of affiliative or agonistic interactions over a given period of time). Regarding network analyses of group living animals, specific SNA have been developed to manage intrinsic biases according to the type of protocol used for data collection (focal sampling, scan sampling, group follow), the heterogeneity of individuals’ observation and the group composition^15, 16^. Given the inherent non-independence of data, the development of different network permutation approaches has permitted the creation of Null Models against which statistical hypotheses can be tested^15^. Although this progress has resulted in a tremendous diversity of SNA software, very few of these software propose an all-in-one toolbox for animal research. Even the most complete software programs, such as UCINET^17^ and SOCPROG^18^, do not provide the most commonly used analysis procedures. Whilst the former lacks data stream permutations, the latter cannot be used for node label permutations and neither software offers time-aggregated SNA or has the high data flexibility of the R environment. R packages, on the other hand, offer high data flexibility, but there is no one package offering an all-in-one toolbox for animal research. For instance, ‘igraph’^19^ and ‘sna’^20^ are among the most used R packages. These are generalist packages (used for all types of network analyses) and therefore do not manage procedures used in animal social network research such as permuted statistical tests. Other packages, such as asnipe^21^, do integrate some data stream permutations but do not allow users to compute SNA measures or run statistical tests. Finally, netTS package allows users to run analyses on time-aggregated networks but does not include the standard procedure for analysing animal networks (node label and data stream permutations). The use of different R packages requires data to be manipulated and/or formatted in specific ways, which requires a certain level of programming knowledge. This switching between the different software packages and the absence of guidelines may cause users to make errors. Hence, these limitations may preclude the use of existing animal social network tools.

**Figure 2:**
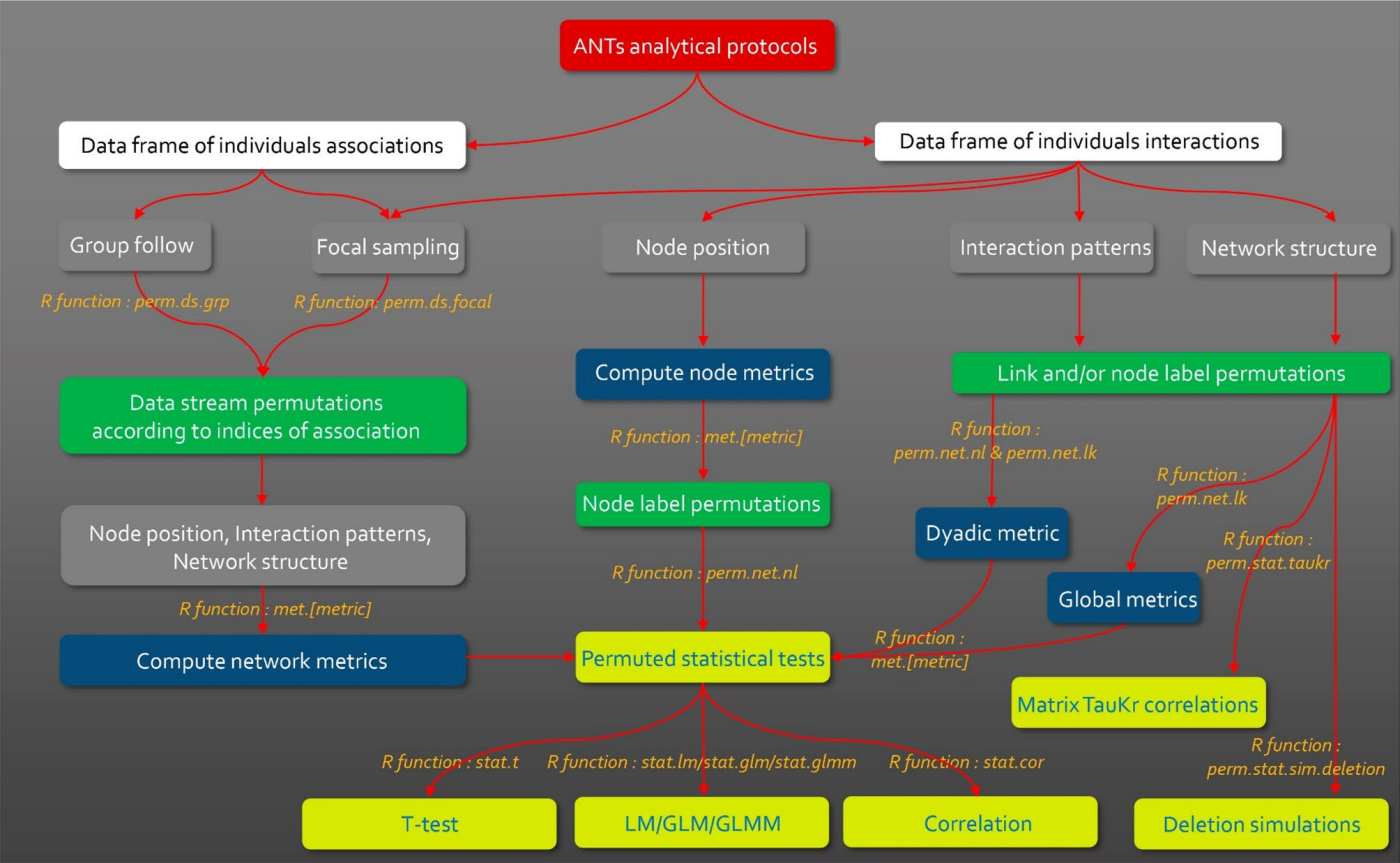
ANTs analytical protocols. In white the types of data handled by ANTs, in gray the theoretical approaches and the types of data collections handled by ANTs, in green the family functions of class ‘perm.’ to perform data permutations, in blue the family functions of class ‘met.’ to compute network measures (node, polyadic or global), in yellow the family functions of class ‘stat’ to perform permuted statistical tests.

The creation of the Animal Network Toolkit software (ANTs) is an attempt to manage these limitations. Whilst ANTs may be of use for all scientists working on social network analyses, it was developed specifically for animal behaviourists and ecologists who want to apply SNA to their research questions with analytical protocols that have been thought, designed and applied for more than 20 years in these specific research areas. This was the main reason behind the publication of this paper and the instructions to use the package (analyses and indices implemented in the packages). This R package has been developed for researchers who are studying animal social networks (usually small networks of up to 1,000 nodes, although the package can be used for larger networks) and have specific observational data protocols. It provides them with a unique software to facilitate the processing of raw data and help researchers to select the most appropriate network measure and permutation approach for their data type, research question and run permuted statistical analyses to perform static and/or temporal (time-aggregated) network analyses. All these steps have been tailored in generic functions based on standard analytical procedures to offer an all-in-one toolbox that is similar to UCINET and SOCPROG, but has the flexibility of the R environment, thus allowing biologist non-expert in graph theory to follow specific analytical protocols, whilst expert users can use ANTs functions to speed up their specific analytical protocols (Fig. 2). Furthermore, ANTs computing speed outperforms ‘igraph’ for the SNA measures that are common to both packages and ‘asnipe’ for data stream permutations (see benchmarks in ESM Appendix 1, Table 1 to Table 6). The efficient and rapid performance of these processes is crucial because some data stream permutation analyses require SNA measures to be computed thousands of times. Finally, ANTs provides multiple variants of a single network measure, as well as R documentation with detailed explanations on these variants and their interpretations. While some of these variants appear in ‘igraph’ and ‘sna’ R packages, others are missing (e.g. Newman 2001 algorithm for shortest paths and related measures: diameter, betweenness, global efficiency). ANTs aims to provide users with an overview of each existing variant measure, thus enabling them to decide which one is the most appropriate for their research question^22^.

**Table 1:** ANTs measures available and their short descriptions.

Measure levels	Measures	Definition
Node	Degree	Number of links
	Outdegree	Number of outgoing links
	Indegree	Number of incoming links
	Strength	Sum of the weights of links
	Outstrength	Sum of the weights of outgoing links
	Instrength	Sum of the weights of incoming links
	R-index	Ratio of outgoing and incoming links
	Disparity	Variation of the weights of the links of a node
	Eigenvector	Node centrality considering the connections of a node and its alters
	Reach	Node centrality considering the overall strength of a node alters
	Affinity	Node centrality considering the overall strength of a node and that of its alters
	Laplacian centrality	Importance of a node in network cohesion
	Betweenness	Number of times a node lies within the shortest paths^a^ of all pairs of nodes in the network
Polyadic	Assortativity^b^	Degree to which a node is linked to others with the same attribute rather than linked to others with a different attribute
	Geodesic distance	Shortest paths^a^ between each pair of nodes in the network
Global	Density	The number of actual connections divided by all potential connections in the network
	Diameter	Greatest shortest path^a^
	Global efficiency	Efficiency of information transmission within the network
	Centralization index	Sum of the differences between the value of the maximum eigenvector centrality with a star network and the eigenvector centrality of each node in the real network’

^a^Shortest distance (based on the number of links or weights of the links) connecting a given pair of nodes.

^b^The calculation of this measure is based on dyadic links but the value returned applies to the whole network.

This paper outlines the large spectrum of analytic opportunities provided by ANTs:

1. Assess data collection reliability.
2. The calculation of the most commonly used and well-known network measures in animal studies as well as other, less well-known measures (Table 1).
3. A multilevel approach through the study of node, polyadic and global network measures.
4. Appropriate randomisation techniques (pre- or network permutations).
5. Several statistical tests (correlation test, t-test, General Linear Model, General Linear Mixed Model) with the calculation of permuted p-values, mean and CI posterior distribution.
6. The possibility to analyse static or temporal networks/comparative analyses for the comparison of different datasets (groups or species).
7. Network visualisation.
8. A fast computing process.

ANTs is currently available in an open beta test version on the following GitHub web page:

https://github.com/SebastianSosa/ANTs (for the stable release)

https://github.com/ants-project/ANTs (for the version under development).

## Methods

### Data input

ANTs can process two types of data: (1) data representing the directed interactions of individuals (e.g. grooming) or their associations (e.g. proximity), and (2) data representing individual attributes (sex, age, dominance rank, etc.).

Interactions and association data can be input in the form of a matrix or a data frame(s). The data frame structure depends on the type of protocol the user wants to follow. For network permutations, data frames must be in an edge list format with at least two columns, one of which indicates the actor and the other the receiver. An additional column may indicate weights of interactions. These data frames allow the user to directly input data collected in the field. For the data stream permutation approach, data can be presented in data frame format. In this case, data frames are not edge lists because they contain additional information in extra columns. For data stream permutations concerning focal observations^16^, i.e. data obtained from following a specific individual over a certain amount of time^23^, two extra columns are required in addition to those indicating the givers and receivers of a behaviour: one indicating the focal individual and another indicating the corresponding focal session. For data stream permutations on group follow observations^6^, i.e. recording individual associations at specific locations and time, data frames have to be in a ‘linear mode’, identical to SOCPROG (i.e. in which each line corresponds to the observation of an individual) with additional columns indicating the different ‘control’ factors (see “Permutations” section) such as the date, time of day or the geographical location associated with the interaction occurred.

It is also possible to use data frames for individual attributes (sex, age, dominance rank, hormone levels, etc.). These must be in a data frame format, with a row for every individual present in the data of individual interactions or associations. Each line represents the attribute(s) of a single individual.

Inputting these two types of data (interactions/associations and individual attributes) may enable the user to (1) permute and/or compute network measures on data representing individuals’ interactions or associations and (2) store node network measures with ANTs functions in the data frame(s) of individual attributes. This makes it possible to study how these node network measures are related to individuals’ attributes.

When performing the multiple networks analytical protocol, the user has to create an R list object where each element of the list stores interaction/association data representing a single network (list of data of interactions or associations). This list must contain a unique data format of interactions/associations (i.e. only edge lists, associations of group follow or associations of focal sampling). Optionally, the user can create a second R list object with the attributes of the individuals present in the corresponding list of interactions/associations (e.g. the data frame of individual attributes in element 1 corresponds to the individuals present in the list of interactions/associations in element 1, etc.). This way, permutations are generated independently in each network (e.g. 1,000 permutations in network 1, 1,000 permutations in network 2, etc.).

### Testing data collection robustness

One of the main issues with regard to social network analysis and the study of animal groups is the quality of data collection (time of observation), as observation biases (e.g. some individuals are more frequently observed than others) can generate unreliable statistical results^24, 25^. Usually, data collection protocol has to be planned for the needs of the intended SNA before collecting data. The following questions must be answered: Do I observe all group members equally? Am I using the best method to limit the disturbance of animal behaviour and interactions? The choice of observation period is also a key factor, as some interindividual associations or interactions are rare and/or difficult to observe over the short term but are still important to attain the objectives of the study. However, this not always the case as scientists often collect data before carrying out analyses. ANTs meets the needs of these differing approaches by offering two different protocols to assess data collection robustness:

1. Lusseau, et al.^24^ protocol to assess the robustness of node measures through bootstrapping.
2. Balasubramaniam, et al.^25^ protocol to assess the robustness of global measures through observation deletion simulations.

For further information on the use of these different protocols, please refer to ANTs R documentation concerning functions in the ‘sampling.’ family.

### Controlling for time heterogeneity

It is sometimes difficult to obtain the same number of observations per individual. ANTs enables users to control for time heterogeneity in different ways through the use of different association indices, namely the generalised affiliation index, the simple ratio index, the half-weight index or the square root index^6^. For further instructions on the use of these different indices, please refer to ANTs R documentation concerning the functions in the ‘assoc.’ family.

### Computing network measures

Three types of network measures can be identified depending on the level of organisation: global measures, polyadic measures, and node measures. In ANTs, all these measures are grouped under the function family ‘*met’*. All the node measures available in ANTs are synthesised in Table 1. The measures we proposed in the package ANTs are the ones commonly used in Animal Social Network Analyses^6, 22, 26–28^.

Global measures (e.g. network diameter) are used to study the overall network and obtain valuable information regarding network efficiency, resilience, clusterisation, etc. Polyadic measures (e.g. assortativity) allow the study of interaction patterns between individuals. These measures provide information about how individuals interact according to their attributes. Node measures (e.g. strength) are the most frequently used measures in animal research. Among other things, node measures inform users about the centrality of an individual, the number of alters it has and/or its activity according to individual attributes, and reveal patterns that are common to individuals with similar attributes. By giving access to global, polyadic and node measures, we aim to enable users to adopt a multilevel approach and thereby understand the centrality of individuals in a group, the patterns of interaction between them and the impact of these two levels on the global network structure^22, 29^ .

For more details on the different types of measures, their mathematical formula, interpretation, limitations and past use in animal research, see Whitehead^6^, Sueur, et al.^26^, Sosa, et al.^22^, Sosa^29^ and refer to ANTs R documentation .

### Permutations

When considering data robustness, permutations can be used to avoid observation biases and ensure the reliability of results obtained by SNA (i.e. results that have no type I and type II errors). Indeed, with the exception of some specific cases such as experiments in social insects, where individuals may be tracked continuously, it is usually assumed when examining inter-individual interactions within a group or a population that neither all the interactions nor all individuals are observed, that the times of observation vary from one individual to another, and that the data collected are intrinsically dependent. For these reasons, permutation tests are needed to control for data independency before performing inferential statistical tests, as inferential statistical tests assume data independency^16^.

The Null Model (NM) approach via permutation is one of the many current possibilities to test statistical hypotheses^15^. It allows users to perform analyses by creating random data sets from the observed data. The observed measure of interest *X* (e.g. coefficient of correlation) is compared to a posterior distribution obtained from the random data sets, and assesses whether *X* is significantly different from the random distribution by calculating the proportion of random values that differ from the observed value. The NM approach can be applied in different ways. ANTs allows for this by adapting the permutations (pre- or network permutations) according to the type of data collected ( i.e. pre- or network permutations for data on associations and interactions respectively) and the research question (i.e. permuting nodes when examining individual network measures or permuting links when examining individual polyadic or global measures).

Data stream and node network permutations are two of the most commonly used permutation methods to build null models in animal social network analysis. A description of these methods is presented by Puga-Gonzalez et al. (submitted). Data stream permutations were initially used to test whether individuals in a social population have a preference for association with certain partners rather than with others^27, 30^. One of the advantages of this method is that it can control for different factors such as location. It is therefore possible to test whether non-random associations are due to individuals’ social preference or result from a preference for the same habitat or location^27^.

Node network permutation is the other commonly used method to test network-related hypotheses in animal research. Node permutations have mainly been used to compare two matrices (or networks) involving the same group of individuals, i.e. matrix correlations. In this case, the values entered in the cell of the matrices are (un)directed behaviours (e.g. grooming or playing). In contrast to the gambit of the group, (un)directed behaviours are usually collected via focal sampling, scan sampling, or ad libitum sampling^23^. During node permutations, the identity of the nodes is redistributed at each permutation whilst the node metric is kept constant. This allows users to test whether a specific network metric is associated with a specific node attribute (e.g. whether females groom more than males), or whether behaviours are reciprocated or directed to individuals with a specific trait (e.g. grooming directed up the dominance hierarchy). All of the permutation approaches available in ANTs are in the family function ‘*perm’* with two subclasses, ‘*perm.ds*’ and ‘*perm.net*’ for data stream and network permutations, respectively. ANTs can perform data stream permutations for group follow and focal sampling data collection protocols. Network permutations can be performed on (1) node label(s) (with labels’ dependency maintained or not), (2) links, (3) link weights, and (4) link weights swap between categories. Among those different types of permutations, node label (ESM Appendix 1) and data stream (ESM Appendix 2) permutations are probably the most commonly used standard approaches in animal network analysis. For this reason, we developed a specific workflow to allow their use (ESM Appendix 1 and ESM Appendix 2) in ANTs for the study of single^31^ or multiple networks^9, 13^ (for network comparisons or time-aggregated analyses). To date, ANTs is the only software permitting the use of these approaches in an all-in-one environment and their application for the analysis of multiple networks.

For more details on the different permutations and their applications according to the data collection protocol, the type of behavioural data collected and the research question, see Bejder, et al.^30^, Whitehead^27^, Whitehead, et al.^32^, Croft, et al.^28^, Farine^16^,Momigliano, et al.^33^, Sosa^29^, ANTs R documentation, ESM Appendix 1 and ESM Appendix 2.

### Statistical tests based on data permutations

All the statistical tests available in ANTs are in the family function ‘stat’. The available tests are correlation test ‘*stat.cor’*, t-test ‘*stat.t*’, Linear Model (LM) ‘*stat.lm*’, Generalised Linear Model (GLM) ‘*stat.glm*’, Generalised Linear Mixed Models (GLMMs) ‘*stat.glmm*’, assortativity test ‘*stat.assortativity*’, TaurK correlation ‘*stat.Taurk*’ and deletion simulation ‘*stat.deletion*’. ANTs *stat.* function returns an object with the posterior distribution of the variable tested.

1. Once the permutation test has been performed, the function ‘ant’, allows the user to obtain the statistical results from any output object of any function ‘*stat’*. The ‘ant’ function returns a data frame with statistics specific to the type of statistical test run. However, some of these statistics are common to all tests, namely the P-values on the right or left of the distribution and the two-side p-values.
2. Measures of the ‘effect size’ of the posterior distribution according to the statistics of interest: 95% confidence interval and the mean of the distribution a posteriori (see Farine and Whitehead^34^). The histograms of the post-distribution of the statistics of interest obtained from the permutations.

### Network visualization

ANTs allows network visualisation with a data frame containing node information and a matrix of interactions/associations. Nodes and links can be parametrised to modify their size and colour and highlight differences (e.g. females showing higher eigenvectors than males). Network layouts are currently based on Barnes Hut repulsion, Hierarchical Repulsion and Force Atlas 2. For more details on network visualisation, see ANTs function ‘*net.vis*’ in the package instructions document. These layouts are commonly used in animal social network analyses^9, 35–37^ as for instance, Force Atlas 2 arranges the visualisation graph with the distance between nodes is inversely proportional to their association, giving a nice view of who is close to whom.

## Discussion

ANTs provides researchers with an all-in-one software containing the most commonly used analytical tools in research on animal social networks. In order to offer tailored functions, ANTs provides what we call analytical protocols that represent standard procedures developed in animal social network analysis over the past decade, namely (1) node label permutations, (2) data stream permutations (for group follow and focal sampling). These two analytical protocols can be used on single networks or time-aggregated networks, allowing unexperienced users to run complex analyses on social networks. Besides, ANTs is also a flexible tool that enables users to conduct each step independently, thus providing users who have programming and analytical skills with an ideal tool to run specific analyses according to their requirements. This will enable users to focus less on coding and more on the selection of the most appropriate permutation approach, statistical test(s) and measures(s), and indeed consider in greater detail why one measure (or version thereof) should be chosen rather than another^22^. ANTs functions ‘which.metric’ and ‘which.protocol’ might be used for an interactive selection. The current version of ANTs includes the most common SNA tools for animal research. New network measures and analytical protocols will be added in the near future with the same objectives: (1) introduce all the variants of each measure, as they are likely to have an impact on the biological interpretations, (2) explain the interest of the measures and protocols depending on the biological question, and (3) optimise the computation speed of the functions. We consider this package to be upgradeable and collaborative and planning to see new indices implemented in ANTs such as those described in these two perspectives papers^18, 38^.

## Supplementary information

Supplementary Information 1.

Supplementary Information 2.

Supplementary Information 3.
