# Supplementary material for: A multilevel statistical toolkit to study animal social networks: the Animal Network Toolkit Software (ANTs) R package
Source: Sci Rep. 2020 Jul 27;10:12507. doi: 10.1038/s41598-020-69265-8 (PMC7385643; doi:10.1038/s41598-020-69265-8)
Supplement: Supplementary file 1 — Supplementary Information 1. [file 41598_2020_69265_MOESM1_ESM.docx]

A multilevel statistical toolkit to study animal social networks: the Animal Network Toolkit Software (ANTs) R package

Sebastian Sosa, Ivan Puga-Gonzalez, Fenghe Hu, Pansanel Jérôme, Xiaohua Xie, Cédric Sueur

***Table 1.*** *Benchmarks for igraph matrix conversion*

***Table 2.*** *Benchmarks for strength, instrength and outstrength functions in R packages ANTs, igraph and sna*

***Table 3.*** *Benchmarks for degree, indegree and outdegree functions in R packages ANTs, igraph and sna*

***Table 4.*** *Benchmarks for betweenness and eigenvector functions in R packages ANTs, igraph and sna*

***Table 5.*** *Benchmarks for diameter, geodesic distance and density functions in R packages ANTs, igraph and sna*

***Table 6.*** *Benchmarks for 1000 pre-network permutations in R packages ANTs and asnipe*

Benchmarks R script

The following benchmarks have been performed with the R package microbenchmark 1.4-6 on a Windows machine, with an AMD Ryzen 7 1700X eight-Core processor (3.40GHz) and with 32Go memory ram. Each benchmark was performed with 100 evaluations. Time units are milliseconds. The computation time does not include matrix conversion to igraph object. The fastest functions are highlighted in bold considering time conversion for igraph functions. The script for benchmarks can be found at the end of the document.

| **Matrix dimension** | **package** | **min** | **mean** | **median** | **max** |
| --- | --- | --- | --- | --- | --- |
| **10*10** | **igraph** | 0.123 | 0.180 | 0.138 | 0.571 |
| **50*50** | **igraph** | 0.236 | 0.359 | 0.294 | 1.080 |
| **100*100** | **igraph** | 0.595 | 0.753 | 0.721 | 1.389 |
| **500*500** | **igraph** | 27.280 | 33.619 | 31.157 | 69.886 |
| **1000*1000** | **igraph** | 134.186 | 166.757 | 151.792 | 428.571 |

***Table 1.*** *Benchmarks for igraph matrix conversion*

|  |  | **Strength** | | | | **Instrength** | | | | **Outstrength** | | | |
| --- | --- | --- | --- | --- | --- | --- | --- | --- | --- | --- | --- | --- | --- |
| **Matrix dimension** | **package** | **min** | **mean** | **median** | **max** | **min** | **mean** | **median** | **max** | **min** | **mean** | **median** | **max** |
| **10*10** | **ANTs** | 0.018 | **0.025** | 0.024 | 0.053 | 0.015 | **0.021** | 0.021 | 0.047 | 0.015 | **0.040** | 0.019 | 2.084 |
|  | igraph | 0.212 | 0.250 | 0.225 | 0.509 | 0.213 | 0.256 | 0.226 | 1.103 | 0.197 | 0.232 | 0.221 | 0.588 |
|  | sna | 0.039 | 0.050 | 0.045 | 0.254 | 0.038 | 0.050 | 0.045 | 0.353 | 0.035 | 0.044 | 0.043 | 0.115 |
| **50*50** | **ANTs** | 0.021 | **0.028** | 0.028 | 0.094 | 0.016 | **0.022** | 0.022 | 0.061 | 0.017 | **0.022** | 0.023 | 0.080 |
|  | igraph | 0.268 | 0.293 | 0.278 | 0.842 | 0.251 | 0.283 | 0.273 | 0.635 | 0.264 | 0.282 | 0.276 | 0.520 |
|  | sna | 0.098 | 0.107 | 0.106 | 0.157 | 0.093 | 0.105 | 0.102 | 0.216 | 0.095 | 0.101 | 0.100 | 0.144 |
| **100*100** | **ANTs** | 0.032 | **0.045** | 0.040 | 0.094 | 0.023 | **0.034** | 0.031 | 0.064 | 0.024 | **0.032** | 0.030 | 0.085 |
|  | igraph | 0.402 | 0.521 | 0.502 | 1.159 | 0.391 | 0.511 | 0.504 | 1.094 | 0.370 | 0.487 | 0.487 | 0.723 |
|  | sna | 0.259 | 0.483 | 0.530 | 0.778 | 0.264 | 0.490 | 0.538 | 0.869 | 0.249 | 0.450 | 0.504 | 0.989 |
| **500*500** | **ANTs** | 1.111 | **1.485** | 1.423 | 3.362 | 0.228 | **0.332** | 0.328 | 0.702 | 0.215 | **0.475** | 0.546 | 0.894 |
|  | igraph | 6.755 | 9.556 | 8.082 | 36.360 | 6.706 | 8.541 | 7.506 | 33.138 | 5.667 | 8.218 | 7.243 | 31.354 |
|  | sna | 9.070 | 16.413 | 11.538 | 206.004 | 8.870 | 14.919 | 11.421 | 41.227 | 8.340 | 14.030 | 11.048 | 39.254 |
| **1000*1000** | **ANTs** | 5.394 | **6.780** | 6.524 | 9.901 | 0.831 | **1.047** | 1.027 | 1.663 | 1.156 | **1.544** | 1.561 | 3.147 |
|  | igraph | 28.181 | 36.820 | 32.924 | 64.742 | 26.212 | 38.552 | 30.218 | 225.424 | 26.424 | 34.993 | 30.114 | 227.700 |
|  | sna | 41.733 | 66.602 | 63.127 | 63.127 | 39.492 | 59.934 | 57.813 | 248.293 | 38.771 | 62.732 | 59.030 | 241.034 |

***Table 2.*** *Benchmarks for strength, instrength and outstrength functions in R packages ANTs, igraph and sna*

|  |  | **Degree** | | | | **Indegree** | | | | **Outdegree** | | | |
| --- | --- | --- | --- | --- | --- | --- | --- | --- | --- | --- | --- | --- | --- |
| **Matrix dimension** | **package** | **min** | **mean** | **median** | **max** | **min** | **mean** | **median** | **max** | **min** | **mean** | **median** | **max** |
| **10*10** | **ANTs** | 0.016 | **0.042** | 0.021 | 1.994 | 0.015 | **0.040** | 0.021 | 1.957 | 0.016 | **0.041** | 0.020 | 2.079 |
|  | igraph | 0.121 | 0.137 | 0.129 | 0.575 | 0.125 | 0.159 | 0.135 | 0.371 | 0.126 | 0.141 | 0.133 | 0.363 |
|  | sna | 0.038 | 0.047 | 0.043 | 0.301 | 0.039 | 0.048 | 0.046 | 0.149 | 0.038 | 0.048 | 0.045 | 0.106 |
| **50*50** | **ANTs** | 0.019 | **0.024** | 0.024 | 0.051 | 0.019 | **0.022** | 0.023 | 0.043 | 0.019 | **0.025** | 0.024 | 0.055 |
|  | igraph | 0.116 | 0.137 | 0.131 | 0.391 | 0.126 | 0.141 | 0.134 | 0.398 | 0.127 | 0.161 | 0.139 | 0.566 |
|  | sna | 0.092 | 0.103 | 0.102 | 0.158 | 0.098 | 0.103 | 0.101 | 0.152 | 0.094 | 0.105 | 0.101 | 0.193 |
| **100*100** | **ANTs** | 0.029 | **0.036** | 0.036 | 0.075 | 0.025 | **0.032** | 0.031 | 0.071 | 0.028 | **0.035** | 0.034 | 0.097 |
|  | igraph | 0.117 | 0.148 | 0.135 | 0.566 | 0.126 | 0.150 | 0.143 | 0.326 | 0.129 | 0.151 | 0.139 | 0.304 |
|  | sna | 0.269 | 0.437 | 0.484 | 0.973 | 0.267 | 0.449 | 0.514 | 0.824 | 0.258 | 0.425 | 0.498 | 0.734 |
| **500*500** | **ANTs** | 0.376 | **0.526** | 0.569 | 0.973 | 0.227 | **0.302** | 0.294 | 0.663 | 0.226 | **0.459** | 0.543 | 1.066 |
|  | igraph | 0.138 | 0.260 | 0.252 | 0.631 | 0.137 | 0.280 | 0.319 | 0.767 | 0.138 | 0.285 | 0.309 | 0.891 |
|  | sna | 9.069 | 13.924 | 11.038 | 40.817 | 8.480 | 14.359 | 11.444 | 38.405 | 8.383 | 14.032 | 11.107 | 39.908 |
| **1000*1000** | **ANTs** | 1.664 | **2.053** | 2.023 | 4.352 | 0.903 | **1.119** | 1.077 | 2.325 | 1.185 | **1.698** | 1.639 | 3.023 |
|  | igraph | 0.135 | 0.336 | 0.362 | 1.370 | 0.156 | 0.318 | 0.353 | 0.586 | 0.148 | 0.371 | 0.370 | 1.721 |
|  | sna | 42.437 | 65.569 | 59.873 | 275.470 | 40.187 | 66.476 | 59.763 | 264.080 | 39.081 | 69.595 | 64.176 | 323.957 |

***Table 3.*** *Benchmarks for degree, indegree and outdegree functions in R packages ANTs, igraph and sna*

|  |  | **Betweenness** | | | | **Eigenvector** | | | |
| --- | --- | --- | --- | --- | --- | --- | --- | --- | --- |
| **Matrix dimension** | **package** | **min** | **mean** | **median** | **max** | **min** | **mean** | **median** | **max** |
| **10*10** | **ANTs** | 0.055 | **0.071** | 0.064 | 0.299 | 0.052 | **0.105** | 0.065 | 3.924 |
|  | igraph | 0.232 | 0.257 | 0.244 | 0.697 | 0.226 | 0.283 | 0.237 | 2.687 |
|  | sna | 0.099 | 0.115 | 0.111 | 0.230 | 0.117 | 0.133 | 0.128 | 0.221 |
| **50*50** | **ANTs** | 0.957 | **1.041** | 1.030 | 1.238 | 0.158 | **0.228** | 0.223 | 0.376 |
|  | igraph | 2.031 | 2.269 | 2.240 | 3.799 | 0.596 | 0.708 | 0.651 | 1.587 |
|  | sna | 4.829 | 5.371 | 5.295 | 7.013 | 1.929 | 2.615 | 2.660 | 10.248 |
| **100*100** | **ANTs** | 4.639 | **4.923** | 4.916 | 5.375 | 0.344 | **0.521** | 0.467 | 1.022 |
|  | igraph | 11.729 | 12.061 | 12.051 | 12.760 | 1.168 | 1.536 | 1.436 | 4.536 |
|  | sna | 34.951 | 41.767 | 37.952 | 332.688 | 7.189 | 9.058 | 8.306 | 19.391 |
| **500*500** | **ANTs** | 267.812 | **293.759** | 284.369 | 391.204 | 5.202 | **18.496** | 7.845 | 483.622 |
|  | igraph | 1034.700 | 1065.964 | 1052.087 | 1735.748 | 29.391 | 78.029 | 50.899 | 499.862 |
|  | sna | 27334.133 | 28581.165 | 28386.552 | 32125.759 | 617.854 | 920.795 | 743.731 | 4239.692 |
| **1000*1000** | **ANTs** | 2597.831 | **2676.158** | 2664.742 | 2903.668 | 35.979 | **65.710** | 41.665 | 742.518 |
|  | igraph | 6814.049 | 6862.835 | 6852.538 | 7005.941 | 297.371 | 338.648 | 314.591 | 1054.610 |
|  | sna | 224515.497 | 232372.981 | 232057.069 | 242910.989 | 3124.892 | 4472.742 | 4504.166 | 6989.336 |

***Table 4.*** *Benchmarks for betweenness and eigenvector functions in R packages ANTs, igraph and sna*

|  |  | **Density** | | | | **Diameter** | | | | **Global efficiency** | | | |
| --- | --- | --- | --- | --- | --- | --- | --- | --- | --- | --- | --- | --- | --- |
| **Matrix dimension** | **package** | **min** | **mean** | **median** | **max** | **min** | **mean** | **median** | **max** | **min** | **mean** | **median** | **max** |
| **10*10** | **ANTs** | 0.010 | **0.026** | 0.013 | 1.146 | 0.015 | **0.026** | 0.020 | 0.417 | 0.010 | **0.014** | 0.012 | 0.083 |
|  | igraph | 0.011 | 0.014 | 0.013 | 0.052 | 0.128 | 0.163 | 0.138 | 1.242 | 0.010 | 0.013 | 0.013 | 0.037 |
|  | sna | 0.045 | 0.058 | 0.053 | 0.335 |  |  |  |  | 0.042 | 0.049 | 0.046 | 0.130 |
| **50*50** | **ANTs** | 0.014 | **0.017** | 0.017 | 0.040 | 0.577 | **0.603** | 0.595 | 0.722 | 0.013 | **0.019** | 0.017 | 0.100 |
|  | igraph | 0.011 | 0.014 | 0.014 | 0.041 | 1.425 | 1.514 | 1.463 | 2.026 | 0.011 | 0.015 | 0.014 | 0.042 |
|  | sna | 0.155 | 0.166 | 0.161 | 0.251 |  |  |  |  | 0.151 | 0.171 | 0.168 | 0.240 |
| **100*100** | **ANTs** | 0.019 | **0.027** | 0.024 | 0.074 | 4.020 | **4.148** | 4.114 | 4.974 | 0.020 | **0.030** | 0.026 | 0.093 |
|  | igraph | 0.010 | 0.018 | 0.015 | 0.094 | 8.321 | 8.798 | 8.650 | 15.067 | 0.011 | 0.019 | 0.017 | 0.087 |
|  | sna | 0.506 | 0.590 | 0.577 | 1.289 |  |  |  |  | 0.492 | 0.593 | 0.566 | 0.891 |
| **500*500** | ANTs | 0.219 | **0.316** | 0.302 | 0.720 | 369.418 | **371.985** | 371.749 | 383.476 | 0.207 | **0.279** | 0.261 | 0.793 |
|  | igraph | 0.014 | 0.053 | 0.049 | 0.145 | 815.697 | 825.364 | 823.269 | 866.273 | 0.013 | 0.041 | 0.039 | 0.145 |
|  | sna | 19.208 | 30.870 | 25.964 | 63.135 |  |  |  |  | 13.358 | 18.181 | 14.834 | 221.123 |
| **1000*1000** | ANTs | 0.889 | **1.083** | 1.042 | 1.647 | 3087.860 | **3163.208** | 3135.216 | 4328.064 | 0.833 | **0.948** | 0.949 | 1.226 |
|  | igraph | 0.013 | 0.052 | 0.051 | 0.279 | 6949.3582 | 7023.271 | 7011.191 | 7247.11 | 0.012 | 0.046 | 0.050 | 0.105 |
|  | sna | 85.715 | 148.057 | 121.003 | 363.356 |  |  |  |  | 63.345 | 108.377 | 75.783 | 275.193 |

***Table 5.*** *Benchmarks for diameter, geodesic distance and density functions in R packages ANTs, igraph and sna*

|  | **Pre-network permutation for group follow** | | | |
| --- | --- | --- | --- | --- |
| **package** | **min** | **mean** | **median** | **max** |
| **ANTs** | 751.871 | **934.891** | 981.476 | 1248.350 |
| asnipe | 2896.433 | 3421.754 | 3339.001 | 5343.831 |

***Table 6.*** *Benchmarks for 1000 pre-network permutations in R packages ANTs and asnipe*

# Benchmarks R script

library(igraph)

library(sna)

library(ANTs)

library(asnipe)

library(microbenchmark)

# data ----------------------------------------------------------------------------------------

m10=matrix(sample(c(0:30),replace = T),ncol = 10,nrow =10)

diag(m10)=0

m50=matrix(sample(c(0:30),replace = T),ncol = 50,nrow =50)

diag(m50)=0

m100=matrix(sample(c(0:30),replace = T),ncol = 100,nrow =100)

diag(m100)=0

m500=matrix(sample(c(0:30),replace = T),ncol = 500,nrow =500)

diag(m500)=0

m1000=matrix(sample(c(0:30),replace = T),ncol = 1000,nrow =1000)

diag(m1000)=0

g10=graph.adjacency(m10,mode = 'directed',weighted = T)

g50=graph.adjacency(m50,mode = 'directed',weighted = T)

g100=graph.adjacency(m100,mode = 'directed',weighted = T)

g500=graph.adjacency(m500,mode = 'directed',weighted = T)

g1000=graph.adjacency(m1000,mode = 'directed',weighted = T)

# Conversion to igraph object ---------------------------------------------------------------

microbenchmark(graph.adjacency(m10,mode = 'directed',weighted = T),

g50=graph.adjacency(m50,mode = 'directed',weighted = T),

g100=graph.adjacency(m100,mode = 'directed',weighted = T),

g500=graph.adjacency(m500,mode = 'directed',weighted = T),

g1000=graph.adjacency(m1000,mode = 'directed',weighted = T) , unit = "ms")

# strength ----------------------------------------------------------------------------------

bench.strength=NULL

bench.strength[[1]]=microbenchmark(ANTs::met.strength (m10),igraph::strength(g10),sna::degree(m10) , unit = "ms")

bench.strength[[2]]=microbenchmark(ANTs::met.strength(m50),igraph::strength(g50),sna::degree(m50) , unit = "ms")

bench.strength[[3]]=microbenchmark(ANTs::met.strength(m100),igraph::strength(g100),sna::degree(m100) , unit = "ms")

bench.strength[[4]]=microbenchmark(ANTs::met.strength(m500),igraph::strength(g500),sna::degree(m500) , unit = "ms")

bench.strength[[5]]=microbenchmark(ANTs::met.strength(m1000),igraph::strength(g1000),sna::degree(m1000) , unit = "ms")

names(bench.strength)=c('mat10','mat50','mat100','mat500','mat1000');bench.strength

# instrength ---------------------------------------------------------------------------------

bench.instrength=NULL

bench.instrength[[1]]=microbenchmark(ANTs::met.instrength(m10),igraph::strength(g10,mode = "in"),sna::degree(m10,cmode="indegree"), unit = "ms")

bench.instrength[[2]]=microbenchmark(ANTs::met.instrength(m50),igraph::strength(g50,mode = "in"),sna::degree(m50,cmode="indegree"), unit = "ms")

bench.instrength[[3]]=microbenchmark(ANTs::met.instrength(m100),igraph::strength(g100,mode = "in"),sna::degree(m100,cmode="indegree"), unit = "ms")

bench.instrength[[4]]=microbenchmark(ANTs::met.instrength(m500),igraph::strength(g500,mode = "in"),sna::degree(m500,cmode="indegree"), unit = "ms")

bench.instrength[[5]]=microbenchmark(ANTs::met.instrength(m1000),igraph::strength(g1000,mode = "in"),sna::degree(m1000,cmode="indegree"), unit = "ms")

names(bench.instrength)=c('mat10','mat50','mat100','mat500','mat1000');bench.instrength

# outstrength --------------------------------------------------------------------------------

bench.outstrength=NULL

bench.outstrength[[1]]=microbenchmark(ANTs::met.outstrength(m10),igraph::strength(g10,mode = "out"),sna::degree(m10,cmode="outdegree"), unit = "ms")

bench.outstrength[[2]]=microbenchmark(ANTs::met.outstrength(m50),igraph::strength(g50,mode = "out"),sna::degree(m50,cmode="outdegree"), unit = "ms")

bench.outstrength[[3]]=microbenchmark(ANTs::met.outstrength(m100),igraph::strength(g100,mode = "out"),sna::degree(m100,cmode="outdegree"), unit = "ms")

bench.outstrength[[4]]=microbenchmark(ANTs::met.outstrength(m500),igraph::strength(g500,mode = "out"),sna::degree(m500,cmode="outdegree"), unit = "ms")

bench.outstrength[[5]]=microbenchmark(ANTs::met.outstrength(m1000),igraph::strength(g1000,mode = "out"),sna::degree(m1000,cmode="outdegree"), unit = "ms")

names(bench.outstrength)=c('mat10','mat50','mat100','mat500','mat1000');bench.outstrength

# degree --------------------------------------------------------------------------------------

bench.degree=NULL

bench.degree[[1]]=microbenchmark(ANTs::met.degree(m10, sym = FALSE),igraph::degree(g10),sna::degree(m10), unit = "ms")

bench.degree[[2]]=microbenchmark(ANTs::met.degree(m50, sym = FALSE),igraph::degree(g50),sna::degree(m50), unit = "ms")

bench.degree[[3]]=microbenchmark(ANTs::met.degree(m100, sym = FALSE),igraph::degree(g100),sna::degree(m100), unit = "ms")

bench.degree[[4]]=microbenchmark(ANTs::met.degree(m500, sym = FALSE),igraph::degree(g500),sna::degree(m500), unit = "ms")

bench.degree[[5]]=microbenchmark(ANTs::met.degree(m1000, sym = FALSE),igraph::degree(g1000),sna::degree(m1000), unit = "ms")

names(bench.degree)=c('mat10','mat50','mat100','mat500','mat1000');bench.degree

# indegree -----------------------------------------------------------------------------------

bench.indegree=NULL

bench.indegree[[1]]=microbenchmark(ANTs::met.indegree(m10),igraph::degree(g10,mode = "in"),sna::degree(m10,cmode="indegree"), unit = "ms")

bench.indegree[[2]]=microbenchmark(ANTs::met.indegree(m50),igraph::degree(g50,mode = "in"),sna::degree(m50,cmode="indegree"), unit = "ms")

bench.indegree[[3]]=microbenchmark(ANTs::met.indegree(m100),igraph::degree(g100,mode = "in"),sna::degree(m100,cmode="indegree"), unit = "ms")

bench.indegree[[4]]=microbenchmark(ANTs::met.indegree(m500),igraph::degree(g500,mode = "in"),sna::degree(m500,cmode="indegree"), unit = "ms")

bench.indegree[[5]]=microbenchmark(ANTs::met.indegree(m1000),igraph::degree(g1000,mode = "in"),sna::degree(m1000,cmode="indegree"), unit = "ms")

names(bench.indegree)=c('mat10','mat50','mat100','mat500','mat1000');bench.indegree

# outdegree ----------------------------------------------------------------------------------

bench.outdegree=NULL

bench.outdegree[[1]]=microbenchmark(ANTs::met.outdegree(m10),igraph::degree(g10,mode = "out"),sna::degree(m10,cmode="outdegree"), unit = "ms")

bench.outdegree[[2]]=microbenchmark(ANTs::met.outdegree(m50),igraph::degree(g50,mode = "out"),sna::degree(m50,cmode="outdegree"), unit = "ms")

bench.outdegree[[3]]=microbenchmark(ANTs::met.outdegree(m100),igraph::degree(g100,mode = "out"),sna::degree(m100,cmode="outdegree"), unit = "ms")

bench.outdegree[[4]]=microbenchmark(ANTs::met.outdegree(m500),igraph::degree(g500,mode = "out"),sna::degree(m500,cmode="outdegree"), unit = "ms")

bench.outdegree[[5]]=microbenchmark(ANTs::met.outdegree(m1000),igraph::degree(g1000,mode = "out"),sna::degree(m1000,cmode="outdegree"), unit = "ms")

names(bench.outdegree)=c('mat10','mat50','mat100','mat500','mat1000');bench.outdegree

# eigenvector ---------------------------------------------------------------------------------

bench.eigenvector=NULL

bench.eigenvector[[1]]=microbenchmark(ANTs::met.eigen(m10),igraph::evcent(g10)$vector,sna::evcent(m10) , unit = "ms")

bench.eigenvector[[2]]=microbenchmark(ANTs::met.eigen(m50),igraph::evcent(g50)$vector,sna::evcent(m50) , unit = "ms")

bench.eigenvector[[3]]=microbenchmark(ANTs::met.eigen(m100),igraph::evcent(g100)$vector,sna::evcent(m100) , unit = "ms")

bench.eigenvector[[4]]=microbenchmark(ANTs::met.eigen(m500),igraph::evcent(g500)$vector,sna::evcent(m500) , unit = "ms")

bench.eigenvector[[5]]=microbenchmark(ANTs::met.eigen(m1000),igraph::evcent(g1000)$vector,sna::evcent(m1000) , unit = "ms")

names(bench.eigenvector)=c('mat10','mat50','mat100','mat500','mat1000');bench.eigenvector

# diameter ---------------------------------------------------------------------------------

bench.diameter=NULL

bench.diameter[[1]]=microbenchmark(ANTs:::metric_global_shortestPath(m10)[[2]],igraph::diameter(g10) , unit = "ms")

bench.diameter[[2]]=microbenchmark(ANTs:::metric_global_shortestPath(m50)[[2]],igraph::diameter(g50) , unit = "ms")

bench.diameter[[3]]=microbenchmark(ANTs:::metric_global_shortestPath(m100)[[2]],igraph::diameter(g100) , unit = "ms")

bench.diameter[[4]]=microbenchmark(ANTs:::metric_global_shortestPath(m500)[[2]],igraph::diameter(g500) , unit = "ms")

bench.diameter[[5]]=microbenchmark(ANTs:::metric_global_shortestPath(m1000)[[2]],igraph::diameter(g1000) , unit = "ms")

names(bench.diameter)=c('mat10','mat50','mat100','mat500','mat1000');bench.diameter

# betweenness ---------------------------------------------------------------------------------

bench.betweenness=NULL

bench.betweenness[[1]]=microbenchmark(ANTs:::metric_node_betweeness(m10),igraph::betweenness(g10),sna::betweenness(m10) , unit = "ms")

bench.betweenness[[2]]=microbenchmark(ANTs:::metric_node_betweeness(m50),igraph::betweenness(g50),sna::betweenness(m50) , unit = "ms")

bench.betweenness[[3]]=microbenchmark(ANTs:::metric_node_betweeness(m100),igraph::betweenness(g100),sna::betweenness(m100) , unit = "ms")

bench.betweenness[[4]]=microbenchmark(ANTs:::metric_node_betweeness(m500),igraph::betweenness(g500),sna::betweenness(m500) , unit = "ms")

bench.betweenness[[5]]=microbenchmark(ANTs:::metric_node_betweeness(m1000),igraph::diameter(g1000),sna::betweenness(m1000) , unit = "ms")

names(bench.betweenness)=c('mat10','mat50','mat100','mat500','mat1000');bench.betweenness

# geodesic distance ---------------------------------------------------------------------------------

bench.geodis=NULL

bench.geodis[[1]]=microbenchmark(ANTs:::metric_global_shortestPath(m10)[[1]],igraph::distances(g10),sna::geodist(m10), unit = "ms")

bench.geodis[[2]]=microbenchmark(ANTs:::metric_global_shortestPath(m50)[[1]],igraph::distances(g50),sna::geodist(m50), unit = "ms")

bench.geodis[[3]]=microbenchmark(ANTs:::metric_global_shortestPath(m100)[[1]],igraph::distances(g100),sna::geodist(m100), unit = "ms")

bench.geodis[[4]]=microbenchmark(ANTs:::metric_global_shortestPath(m500)[[1]],igraph::distances(g500),sna::geodist(m500), unit = "ms")

bench.geodis[[5]]=microbenchmark(ANTs:::metric_global_shortestPath(m1000)[[1]],igraph::distances(g1000),sna::geodist(m1000), unit = "ms")

names(bench.geodis)=c('mat10','mat50','mat100','mat500','mat1000');bench.geodis

# density ---------------------------------------------------------------------------------

bench.density=NULL

bench.density[[1]]=microbenchmark(ANTs::met.density(m10),igraph::graph.density(g10),sna::gden(m10), unit = "ms")

bench.density[[2]]=microbenchmark(ANTs::met.density(m50),igraph::graph.density(g50),sna::gden(m50), unit = "ms")

bench.density[[3]]=microbenchmark(ANTs::met.density(m100),igraph::graph.density(g100),sna::gden(m100), unit = "ms")

bench.density[[4]]=microbenchmark(ANTs::met.density(m500),igraph::graph.density(g500),sna::gden(m500), unit = "ms")

bench.density[[5]]=microbenchmark(ANTs::met.density(m1000),igraph::graph.density(g1000),sna::gden(m1000), unit = "ms")

names(bench.density)=c('mat10','mat50','mat100','mat500','mat1000');bench.density

# Pre-network permutations---------------------------------------------------------------------

# extracting data from the package asnipe

data ("group by individual")

data("times")

# Converting “group by individual” matrix into a data frame for ANTs functions

row.names(gbi) = 1:nrow(gbi)

colnames(gbi) = 1:ncol(gbi)

df = ANTs:::gbi.to.df(gbi)

# Benchmark

Pre-network = microbenchmark(perm.ds.grp(df, scan = 'scan', perm = 1000),

network permutation(gbi, permutations = 1000)
