# Supplementary material for: A multilevel statistical toolkit to study animal social networks: the Animal Network Toolkit Software (ANTs) R package
Source: Sci Rep. 2020 Jul 27;10:12507. doi: 10.1038/s41598-020-69265-8 (PMC7385643; doi:10.1038/s41598-020-69265-8)
Supplement: Supplementary file 2 — Supplementary Information 2. [file 41598_2020_69265_MOESM2_ESM.docx]

A multilevel statistical toolkit to study animal social networks: the Animal Network Toolkit Software (ANTs) R package

Sebastian Sosa, Ivan Puga-Gonzalez, Fenghe Hu, Pansanel Jérôme, Xiaohua Xie, Cédric Sueur

**Examining node level in static or time-aggregated networks with R package ANTs through node label permutations**

*Abstract*: Animal Network Toolkit software (ANTs) is an R package designed to perform social network analysis of animal social systems. One of the goals of this package is to provide users with tools that enable them to correctly use of their data; facilitate the use of complex analytical approaches. Here, we provide a tutorial to perform social networks analysis at the individual (node) level, in static or time-aggregated networks, with ANTs. ANTs’ workflow is based on two different objects: adjacency matrices (representing individual associations or interactions) and data frames (containing individuals’ characteristics such as sex, age, hierarchical rank, etc.). Adjacency matrices are used to calculate network node metrics and the data frames to store calculated data and perform node label permutations and statistical tests. The tutorial is divided into five different sections: 1) converting raw data into adjacency matrices, 2) computing network measures, 3) performing permutations, 4) replicating statistical tests on permutations, and 5) computing statistics on the posterior distribution of these permutation tests.

# Introduction

Node label permutations is a useful approach when studying individual metrics and when the data collected concern contact or interaction behaviors (e.g. grooming, embracing, playing, etc.), in which the level of uncertainty is low ([Croft *et al.* 2011](#_ENREF_3)).The following tutorial provides explanations on how to analyze single ([Sosa 2016](#_ENREF_7); [Liao *et al.* 2018](#_ENREF_6)) or multiple network(s) ([Borgeaud *et al.* 2017](#_ENREF_2); [Sosa *et al.* 2018a](#_ENREF_9); [Kawazoe & Sosa 2019](#_ENREF_5)) with the R package [Animal Network Toolkit software (ANTs)](http://www.s-sosa.com/ants) ([Sosa *et al.* 2018b](#_ENREF_10)) using node label permutations ([Sosa 2018](#_ENREF_8)). Single network analyses are available in Ucinet software or asnipe R package, for example. However, multiple networks analyses are not available in any Social Network Analysis (SNA) software to our knowledge. ANTs is a free open R package for the analysis of animal networks. We made an effort to provide users with numerous tools from graph theory to perform statistical analyses for testing hypotheses.

We will start with a data frame of interactions but it is possible to start with an adjacency matrix representing the network of interactions. The data frame format is described in [1.1. Starting with a data frame of interactions](#section1) section. All ANTs analytical protocols use an adjacency matrix (representing individual interactions) and a data frame in a supplementary file (representing individual characteristics such as sex, age, hierarchical rank, etc.). The adjacency matrix is used to compute common network node metrics and the data frame stores these network node metrics, on which the permuted statistical test will be run. All ANTs functions detect if the data input is a data frame (for a single network protocol) or a list of data frames (for multiple networks protocol), so the same functions can be used for analyzing a single network or multiple networks. These are the different steps to run these two protocols from raw data to permuted statistical results: 1) [Import data of interactions](#_2._Import_data), 2) [Import (a) data frame(s) of individual characteristics](#_3._Import_a), 3) [Compute node metrics](#_4._Compute_node), 3) [Perform node label permutations](#_5._Perform_node), 4) [Permuted statistical test and results](#_6._Run_permuted).

# 1. Install ANTs

Currently ANTs is not available in CRAN but only in GitHub and must be installed via R package *devtools* with the following command

devtools::install_github('SebastianSosa/ANTs')

# 2. Import data of interactions

Start by calling ANTs in your R environment.

library(ANTs)

You can import two types of data of interactions: 1) An edge list of interactions or 2) An adjacency matrix of interactions. As raw data are usually stored in an edge list of interactions, we will start with the tutorial corresponding to this type of data. In ANTs, an edge list of interactions must comprise at least two columns:

1. A column indicating the id of the individual performing the behavior

2. A column indicating the id of the individual receiving the behavior

A third column can also be present if the interactions collected are weighted (*e.g.* duration of grooming). This will be taken into account when converting the edge list into an adjacency matrix of interactions.

In this tutorial, we simulated the interactions of a group of individuals that are characterized by their ID, their age and sex. In Appendix 4, you can download a zip file in which data for simulated single networks and multiple networks can be found.

## 2.1. Import a single data frame of interactions

To import a single data frame of interactions, use R function *df=read.csv(...)*.

In Appendix 4 in the following directory you can find the simulated interactions for a single network: ‘single network/node label/interactions.csv’. Set your working directory and import the data.

setwd("Your.Path...tutoriels/single network/node label")
d = read.csv(file = 'interactions.csv', header = TRUE)

Here is an example of a data frame of interactions without the weights’ column:

head(d)

| ## | **focal** | **alter** |  |
| --- | --- | --- | --- |
| ## | N | H |  |
| ## | N | C |  |
| ## | I | B |  |
| ## | C | N |  |
| ## | M | G |  |
| ## | O | I |  |

## 2.2. Import multiple data frames of interactions

When studying a group over several periods and/or when studying several groups (of the same or different species), one obtains several independent networks in which node label permutations must be run separately. To do so, ANTs needs to store the edge list in a list format. Each of the edge list has to be structured in the same way, explained in section [2.1. Import a single data frame of interactions](file:///C:\Users\Sebastian\Documents\ANT%20unofficial\tutoriels\section1). To analyze this type of data, ANTs allows to run permuted Generalized Linear Mixed Models where the different networks are permuted separately (from now on, these factors will be referred to as random factors).

In this tutorial, we simulated the interactions of two groups of individuals that are characterized by their IDs and also by their age and sex. To import several data frames of interactions at once, use the ANTs function *import.df* once you have changed the R working directory in which you saved all the data frames in separate *.csv* files without any other file. *import.df* function imports all the files present in the R working directory in the form of a list, each element of the list named according to the corresponding ‘.csv’ file and ordered alphabetically. import.df function has the same arguments as R function read.csv and thus can handle different separators, coma formats, files with headers and row names, etc. (for more information, type *?read.csv* or *?import.df*).

In Appendix 4, in the following directory you can find the simulated interactions for multiple networks: ‘multiple networks/Node label permutations/interactions’ with two csv files ‘group1.csv’ and ‘group2.csv’. Set the working directory and import data frames of interactions:

setwd("Your.Path.../tutoriels/multiples groups/Node label permutations/interactions")
ldf = import.df(sep = ";")

## 2.3. Convert a data frame of interactions into an adjacency matrix

To convert a data frame of interactions into an adjacency matrix, use the function *df.to.mat* in which you have to state at least the edge list or list of edge lists (argument *df*) and the column numbers or names holding the ‘actor’ (*i.e.* the individual emitting the behaviour for directed interactions) (argument *actor*) and the ‘receiver’ (*i.e.* the individual receiving the behavior for directed interactions) (argument *receiver*).

If the behavior is undirected, and if you want to symmetrize the matrix, the argument *sym* allows to convert the data frame of interactions into a symmetric matrix by considering that a single interaction between two individuals is an incoming and outgoing tie for both individuals.

If the interactions are weighted (*e.g.* duration data), the weights of the interactions have to appear in a specific column stated with the argument *weighted* as an integer or string indicating the column in which the weights of the interactions are listed. If interaction weights are null, each row is assigned a weight of one. However, note that if two individuals interact with each other more than once, and this is specified in the data frame with two row entries, then a weighted matrix is generated as output. The matrix generated is arranged according to the order of appearance of individuals in the edge list of interactions. Thus, future data manipulations with ANTs require to consider the order of individuals between the generated matrix and the data frame of individual characteristics.

In order to control for heterogeneity in observation time per individual, ANTs allows you to state argument *tobs* as a numeric vector holding each individual’s time of observation. It has to be ordered alphabetically by id names (the same names as in df$actor and df$receiver columns). If IDs are numbers, state argument *num.ids* must be equal to *TRUE*. When heterogeneity in observation time is stated, each interaction between individuals is divided by the sum of the times of observation of the actor and the receiver (i.e. interactions between *A* and *B* / time of observation of *A* and *B*).

### 2.3.1. Convert a single data frame

m = df.to.mat(d, actor = 1, receiver = 2,weighted = NULL, sym = F, tobs = NULL, num.ids = FALSE)
m

| **##** | **A** | **B** | **C** | **D** | **E** | **F** | **G** | **H** | **I** | **J** | **K** | **L** | **M** | **N** | **O** | **S** | **Y** |
| --- | --- | --- | --- | --- | --- | --- | --- | --- | --- | --- | --- | --- | --- | --- | --- | --- | --- |
| ## **A** | 0 | 0 | 1 | 0 | 0 | 1 | 0 | 0 | 0 | 0 | 2 | 0 | 0 | 1 | 0 | 0 | 0 |
| ## **B** | 0 | 0 | 1 | 2 | 0 | 0 | 0 | 0 | 1 | 1 | 1 | 0 | 0 | 0 | 0 | 0 | 0 |
| ## **C** | 0 | 0 | 0 | 0 | 0 | 1 | 1 | 1 | 0 | 0 | 1 | 0 | 1 | 3 | 0 | 0 | 0 |
| ## **D** | 0 | 0 | 0 | 0 | 0 | 1 | 0 | 0 | 1 | 1 | 0 | 2 | 0 | 2 | 0 | 0 | 0 |
| ## **E** | 2 | 0 | 2 | 0 | 0 | 1 | 0 | 0 | 1 | 0 | 0 | 1 | 0 | 0 | 1 | 0 | 0 |
| ## **F** | 0 | 0 | 0 | 0 | 0 | 0 | 0 | 1 | 1 | 0 | 0 | 0 | 0 | 0 | 0 | 0 | 0 |
| ## **G** | 1 | 1 | 0 | 0 | 0 | 0 | 0 | 1 | 0 | 1 | 2 | 0 | 0 | 0 | 0 | 0 | 0 |
| ## **H** | 1 | 2 | 0 | 0 | 0 | 1 | 0 | 0 | 0 | 0 | 0 | 0 | 0 | 0 | 0 | 0 | 0 |
| ## **I** | 0 | 1 | 0 | 1 | 0 | 0 | 1 | 0 | 0 | 1 | 0 | 0 | 1 | 0 | 0 | 0 | 0 |
| ## **J** | 0 | 0 | 1 | 0 | 1 | 2 | 0 | 0 | 0 | 0 | 2 | 0 | 1 | 0 | 1 | 0 | 0 |
| ## **K** | 1 | 0 | 1 | 0 | 0 | 1 | 1 | 1 | 0 | 0 | 0 | 0 | 0 | 0 | 0 | 1 | 0 |
| ## **L** | 0 | 0 | 0 | 1 | 1 | 1 | 0 | 1 | 0 | 0 | 0 | 0 | 1 | 1 | 0 | 0 | 0 |
| ## **M** | 1 | 0 | 2 | 1 | 0 | 0 | 2 | 0 | 1 | 0 | 0 | 1 | 0 | 0 | 0 | 0 | 0 |
| ## **N** | 0 | 0 | 2 | 0 | 1 | 1 | 0 | 1 | 3 | 0 | 1 | 3 | 1 | 0 | 0 | 0 | 0 |
| ## **O** | 1 | 1 | 0 | 1 | 0 | 0 | 0 | 1 | 1 | 0 | 0 | 0 | 1 | 1 | 0 | 0 | 1 |
| ## **S** | 0 | 0 | 0 | 0 | 0 | 0 | 0 | 0 | 0 | 0 | 0 | 0 | 0 | 0 | 0 | 0 | 0 |
| ## **Y** | 0 | 0 | 0 | 0 | 0 | 0 | 0 | 0 | 0 | 0 | 0 | 0 | 0 | 0 | 0 | 0 | 0 |

## attr(,"ANT")
 ## [1] "Matrix obtained through a data frame of interactions"

This function returns a matrix of dimensions equal to the unique ids contained in both ‘actor’ and ‘receiver’ columns and ordered alphabetically for strings or in increasing order for numbers.

### 2.3.2. Convert multiple data frames

Ml = df.to.mat(ldf, actor = 1, receiver = 2, weighted = NULL, sym = FALSE)

This function returns a list of matrices of dimensions equal to the unique ids contained in both ‘actor’ and ‘receiver’ columns of the data frame corresponding to the list of data frames, and ordered alphabetically for strings or in increasing order for numbers.

## 2.4. Start with a matrix

If you want to start directly with a matrix, simply use the R function *m=as.matrix(read.csv(...))* for a single matrix or the ANTs function *import.mat* to import matrices. ANTs function *import.mat* works exactly the same way as the ANTs function *import.df* explained in section [2.2. Importing multiple data frames of interactions](#_2.2._Import_multiple).

N.B. This requires to control first for the time of observation per individual and dyad.

# 3. Import a data frame of individual characteristics

ANTs works simultaneously with two different objects, the adjacency matrix(ces) (representing individual interactions) and the data frame(s) (representing individual characteristics such as sex, age, hierarchical rank, etc.). The adjacency matrix(ces) is (are) used to compute network node metrics and the data frame stores these metrics. The data store in the data frame is later used to perform node label permutations and statistical tests.

## 3.1. Import a single data frame of individual characteristics

As for importing a data frame of interactions, use the R function *df=as.data.frame(read.csv(...))*.

In Appendix 4 in the following directory you can find the simulated individual characteristics for a single network: ‘single network/node label/characteristics.csv’. Set your working directory and import the data.

setwd("Your.Path.../tutoriels/single network/node label")
di = read.csv(file = 'characteristics.csv', header = T)

Example of data frame of individuals characteristics:

head(di)

| **##** | **id** | **sex** | **age** |  |
| --- | --- | --- | --- | --- |
| ## | E | F | 14 |  |
| ## | I | M | 15 |  |
| ## | F | F | 11 |  |
| ## | G | M | 17 |  |
| ## | K | F | 15 |  |
| ## | J | M | 15 |  |

If you do not have a data frame of individual characteristics, ANTs allows you to create an empty data frame the same size as the matrix with the function *df.create(...)*.

## 3.2. Import multiple data frames of individual characteristics

Like for importing multiple data frames of individual interactions, use the ANTs function *import.df* to import multiple data frames of individual characteristics. These files must be in a separate folder, from those of the edge list (or matrices) of interactions, and must be named identically to the files gathering the interactions, because the function *import.df* imports the files following an alphabetical order. Each data frame must hold a column(s) indicating the factor(s) that identifies each network (in this case, the column group).

In Appendix 4 in the following directory you can find the simulated individual characteristics for multiple networks: ‘multiple networks/Node label permutations/characteristics’ with two csv files ‘group1.csv’ and ‘group2.csv’. Select working directory and import your data frames of individual characteristics:

setwd("Your.Path.../tutoriels/multiples groups/Node label permutations/characteristics")
ldi = import.df(sep = ";")

If you do not have a data frame of characteristics, ANTs allows you to create an empty data frame the same size as the matrix with the function *df.create(...)*.

# 4. Compute node measures

Now you have the adjacency matrix and the data frame. ANTs allows you to compute several node measures (for an overview of them see [Sosa (2018)](#_ENREF_8)). All of them belong to the type met.:

1. Degree (*met.degree*)
2. Indegree (*met.indegree*)
3. Outdegree (*met.outdegree*)
4. Strength (*met.strength*)
5. Instrength (*met.instrength*)
6. Outstrength (*met.outstrength*)
7. Eigenvector (*met.eigenvector*)
8. Laplacian centrality (*met.lp*)
9. Reach (*met.reach*)
10. Disparity (*met.disparity*)
11. Affinity (*met.affinity*)
12. R-I index (*met.ri*)
13. Betweenness (*met.betweenness*)

ANTs Graphical User Interface allows you to compute all these node measures using the function *met*.

## 4.2. Possible outputs

ANTs’ *met* functions take a matrix(ces) as main argument. The functions detect if this input is a single matrix (for a single network protocol) or a list of matrices (for a multiple networks protocol). Thus the same functions can be used to compute any of the network metrics present in ANTs. The output of the *met* functions can be either a vector with the values of each node metric:

met.degree(m)

## A B C D E F G H I J K L M N O S Y
## 9 8 10 8 8 10 8 9 9 10 9 6 9 11 10 1 1

met.degree(ml)

## $group1
## A B C D E F G H I J K L M N O Z
## 6 13 8 12 10 7 11 11 8 12 7 9 9 9 7 1
##
## $group2
## a b c d e f g h i j k l m n o p u
## 8 9 11 10 7 8 7 11 12 10 8 6 14 7 10 1 1

or a data frame, in which one of the columns will contain the node measure calculated by the *met* function. For the data frame output, the argument *df* of the *met* function should be a data frame with at least one column containing the individuals’ IDs. Note, that rows’ and columns’ names of the input matrix have to be in the same order as the IDs’ column of the input data frame (this may be the case when you start with an edge list of interactions because the matrix generated is arranged according order of apparition of individuals in the edge list of interaction). To order the data frame according to the matrix(ces) you can use the argument *dfid* of the *met* function to indicate the IDs’ column of the input data frame. In this way, ANTs will match the IDs of the input data frame to the IDs of the input matrix and create a column in the output data frame with the node measure in that specific ID order. Note that to perform permuted statistical tests, node metrics must be stored in the data frame.

met.degree(m, df = di, dfid = 1)

| **##** | **id** | **sex** | **age** | **degree** |  |
| --- | --- | --- | --- | --- | --- |
| ## | A | F | 20 | 9 |  |
| ## | B | F | 15 | 8 |  |
| ## | C | M | 14 | 10 |  |
| ## | D | F | 19 | 8 |  |
| ## | E | F | 14 | 8 |  |
| ## | F | F | 11 | 10 |  |
| ## | G | M | 17 | 8 |  |
| ## | H | F | 12 | 9 |  |
| ## | I | M | 15 | 9 |  |
| ## | J | M | 15 | 10 |  |
| ## | K | F | 15 | 9 |  |
| ## | L | M | 18 | 6 |  |
| ## | M | F | 1 | 9 |  |
| ## | N | F | 1 | 11 |  |
| ## | O | F | 10 | 10 |  |
| ## | S | M | 7 | 1 |  |
| ## | Y | F | 16 | 1 |  |

Now, we will store this node metric in the data frame of individual characteristics to perform node label permutations by declaring:

- 1. *df*, the argument specifying the data frame in which to perform node label permutations
  2. *dfid* and optional argument, to declare which column of the data frame stores the individuals’ IDs, which will help ANTs function to merge the node metric with the corresponding individuals in the data frame.

df = met.degree(m, df = di, dfid = 1)

We will do the same for the list of data frames.

Ldi = met.degree(ml, df = ldi, dfid = 1)

# 5. Perform node label permutations

Node label permutations is a useful approach when the data collected in which the level of uncertainty is low ([Croft *et al.* 2011](#_ENREF_3); [Sosa 2018](#_ENREF_8)), such as contact behaviors (e.g. grooming, embracing, playing, etc.). Here, the null hypothesis is to test if nodes’ metrics are randomly distributed according to individual node labels against hypotheses one may be interested in, for instance, whether a node metric is correlated with an individual characteristic (sex, age, dominance rank, etc.). To perform node label permutations with ANTs, use the function *perm.nl*. This function adapts the permutation process according to the type of input specified in the argument *df*. If the argument *df* is a single data frame, then node label permutations are performed within the data frame. If argument *df* is a list of data frames, then the permutations are performed within each data frame and after each permutation a new data frame is created by merging all the permuted data frames. This process is repeated as many times as define in the argument *nperm*. When loading a list of data frames for multiple networks protocol, an additional argument rf (random factors) has to be declared, indicating which column(s) is (are) the random factor(s) (*e.g.* different groups and/or different periods of group observations).

In addition, *perm.nl* function comprises the following arguments:

1. *df* is a data frame that holds nodes’ information.
2. *labels* is a numeric/integer vector or character vector. If the argument labels is a numeric or integer vector, it indicates the column(s) number(s) to permute. If the argument labels is a character vector, it indicates the column(s) name(s) to permute.
3. *rf* is a numeric/integer vector or character vector. If the argument rf is a numeric or integer vector, it indicates the column(s) number(s) of the random factors. If the argument rf is a character(s) vector, it indicates the column(s) name(s) that identifies each network.
4. *nperm* is an integer that indicates the number of permutations to perform.
5. *progress* is a boolean (TRUE or FALSE) that indicates the progression of the permutation process. By default, this option is equal to TRUE. However, this option may slow down the permutation process.

This function returns a list of data frames of length *nperm+1*.

## 5.1 Node label permutations for single network

pd = perm.net.nl(di, labels = c('sex', 'age'), nperm = 1000, progress = TRUE, rf = NULL)

## 5.2 Node label permutations for multiple networks

pld = perm.net.nl(ldi, labels = c('sex', 'age'), nperm = 1000, progress = TRUE, rf = 'group')

# 6. Run permuted statistical tests & compute permuted statistical results

ANTs allows to run the following statistical tests on permuted data using the functions of type *stat.*:

1. Correlation
2. T-test
3. LM/GLM
4. GLMM
5. Assortativity test
6. TauKr test
7. Deletion simulations

In this tutorial, we will only see how to perform permuted [correlation tests](#section5.3.1), [t-tests](#section5.3.2) and [LM test](#section5.3.3), [GLM test](#section5.3.4) and [Generalized Linear Mixed Models](#section5.4) (GLMM) as we are currently working at a node level.

## 6.1. ANTs statistical result

ANTs allows to run a diagnostic test from permuted statistics obtained through the functions of type *stat*. with the method *ant*. This method adapts the diagnostic results according to the data input. The output is adapted to the type of test run. However, some outputs are common to all tests, a data frame of statistics of permutation tests:

1. P-values, by the nature of the test, they are one-tailed p-values, on the right and left of the distribution.
2. Measurement of the effect size of the post-distribution according to the statistics of interest ([Farine & Whitehead 2015](#_ENREF_4)), 95% confidence interval, and mean.
3. A histogram of the post-distribution of the statistics of interest with the value of the statistics of interest for the real data highlighted in white.

## 6.2 Single network analysis

### 6.2.1 Correlation

We will start with a simple correlation between age and degree with the function *stat.cor*.
Based on R *cor* function, *stat.cor* handles the same arguments, consequently allowing Pearson, Spearman and Kendall correlations. *stat.cor* has two extra arguments:

1. *ant* for the permuted data
2. progress to print the progression of the analysis

C=stat.cor(ant = pd, 'age', 'degree', method = 'pearson', progress = TRUE)
c=ant(C)

The *ant* function returns a list of two elements:

For correlation test, the *ant* function returns an object with:

1. A summary of the statistical test with the data frame of statistics of permutation test:

c$statistics

|  | **Observed correlation** | **p.left** | **p.right** | **p.one.side** | **95ci lower** | **95ci upper** | **mean** |
| --- | --- | --- | --- | --- | --- | --- | --- |
| **statistics** | -0.122 | 0.398 | 0.597 | 0.796 | -0.025 | 0.0065 | -0.009 |
|  |  |  |  |  |  |  |  |

1. A histogram of the post-distribution of the statistics of interest with the value of the statistics of interest for the observed data highlighted in white:

c$post.dist


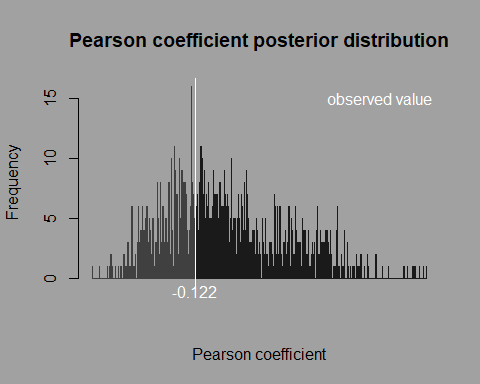


### 6.2.2 T-test

We will continue with a t-test to test for sex differences in the metric degree with the function *stat.t.* This function is based on the R function *t.test*.

*stat.t* handles the same arguments as in the *s3* formula method, with two extra arguments:

1. *ant* for the permuted data
2. *progress* to print the progression of the analysis

TT = stat.t(ant = pd, formula = degree ~ sex, progress = TRUE)
t = ant(TT)

For T-test, the *ant* function returns an object of two elements:

1. A summary of the statistical test with the data frame of statistics of permutation test:

t$statistics

|  | **t observed** | **p.left** | **p.right** | **p.one.side** | **95ci lower** | **95ci upper** | **mean** |
| --- | --- | --- | --- | --- | --- | --- | --- |
| **statistics** | 0.6389 | 0.725 | 0.257 | 0.514 | -0.216 | -0.075 | -0.145 |

1. A histogram of the post-distribution of the statistics of interest with the value of the statistics of interest for the observed data highlighted in white:

t$post.dist


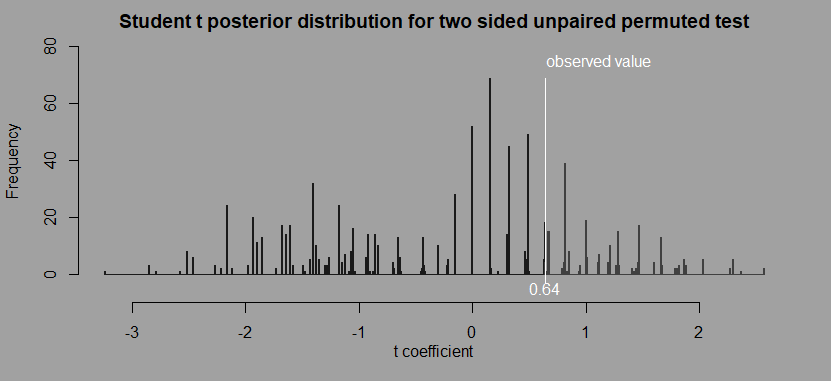


### 6.2.3 LM

Now we will run a linear model to simultaneously test for age and sex differences with the function *stat.lm*.

Based on R *lm* function, *stat.lm* handles the same arguments with 2 extra arguments:

1. *ant* for the permuted data
2. *progress* to print the progression of the analysis

LM = stat.lm(ant = pd,formula = degree ~ age + sex, progress= TRUE)
l = ant(LM)

For linear models, the *ant* function returns an object with three elements:

1. A summary of the original LM model with the data frame of statistics of permutation test:

l$model

## Call:
## "degree ~ age + sex"
##
## Residuals:
## Min 1Q Median 3Q Max
## -7.1829 -0.2302 0.7698 1.5804 2.6982
##
## Coefficients:
## Estimate Std. Error t value p.left p.rigth p.one.side
## (Intercept) 8.940415 1.914585 4.669636 0.699700 0.300300 0.600601
## age -0.047348 0.138392 -0.342126 0.340340 0.659660 0.680681
## sexM -0.928434 1.556245 -0.596586 0.122122 0.877878 0.244244
## lower.ci uper.ci mean
## (Intercept) 8.177874 8.403378 8.291
## age -0.003294 0.013869 0.005
## sexM -1.042940 -0.991689 -1.017
##
## Residual standard error: 3.01 on 14 degrees of freedom
## Multiple R-squared: 0.03925, Adjusted R-squared: -0.098
## F-statistic: 0.286 on 2 and 14 DF, p-value: 0.7555

1. Two plots of diagnostic of the original model:

l$model.diagnostic


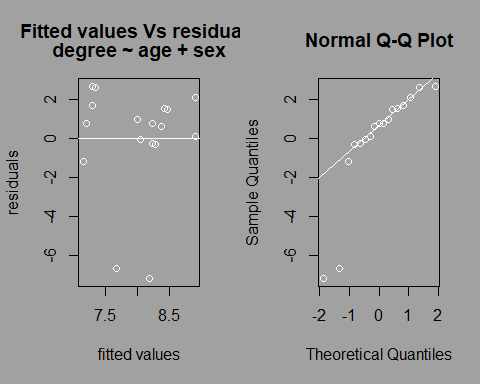


1. A histogram of the post-distribution of the statistics of interest with the value of the statistics of interest for the real data highlighted in white:

l$post.dist


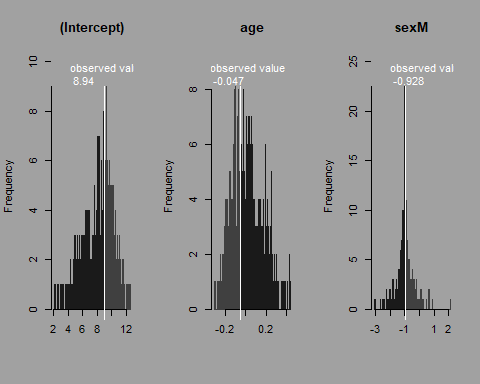


### 6.2.4 GLM

Similar analyses can be done with generalized linear models with different types of family distribution.
Based on R *glm* function, *stat.glm* handles the same arguments with 2 extra arguments:

1. *ant* for the permuted data
2. *progress* to print the progression of the analysis

GLM = stat.glm(ant = pd,formula = degree ~ age + sex, progress = TRUE, family = 'gaussian')
glm = ant(GLM)

For GLM, the *ant* function returns an object of three elements:

1. A summary of the original GLM model with the data frame of statistics of permutation test:

glm$model

##
## Call:
## "degree ~ age + sex , family = gaussian"
##
## Deviance Residuals:
## Min 1Q Median 3Q Max
## -7.1829 -0.2302 0.7698 1.5804 2.6982
##
## Coefficients:
## Estimate Std. Error t value p.left p.rigth p.one.side## (Intercept) 8.940415 1.914585 4.669636 0.699700 0.300300 0.600601
## age -0.047348 0.138392 -0.342126 0.340340 0.659660 0.680681
## sexM -0.928434 1.556245 -0.596586 0.122122 0.877878 0.244244
## lower.ci uper.ci mean
## (Intercept) 8.177874 8.403378 8.291
## age -0.003294 0.013869 0.005
## sexM -1.042940 -0.991689 -1.017
##
## (Dispersion parameter for gaussian family taken to be 9.058464)
##
## Null deviance: 132.00 on 16 degrees of freedom
## Residual deviance: 126.82 on 14 degrees of freedom
## AIC: 90.406
##
## Number of Fisher Scoring iterations: 2

1. Two plots of diagnostic of the original model:

glm$model.diagnostic


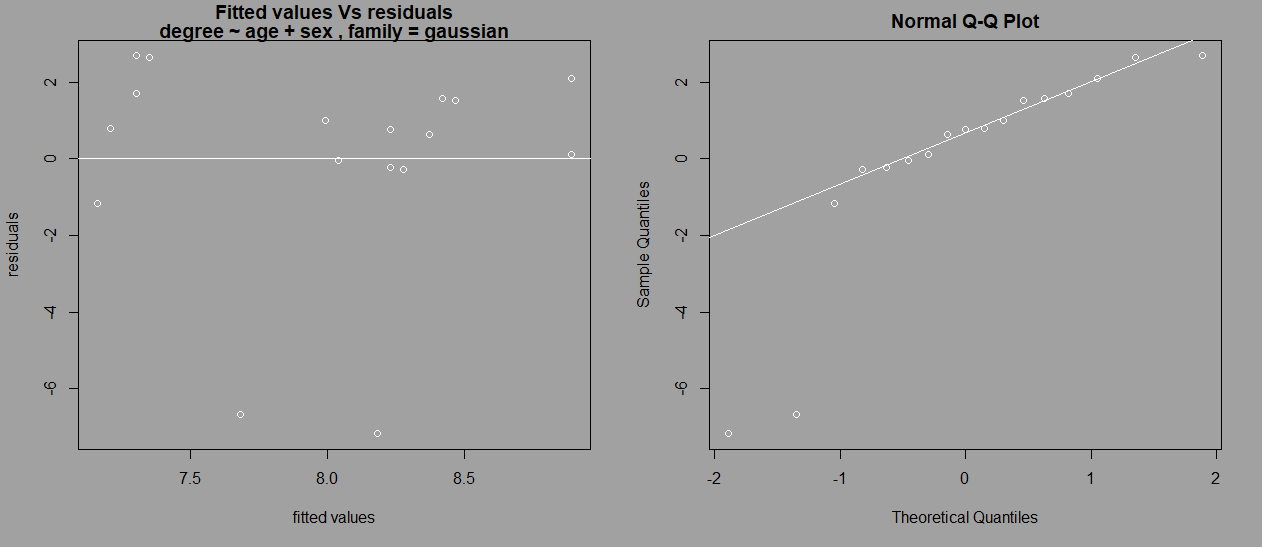


1. A histogram of the post-distribution of the statistics of interest with the value of the statistics of interest for the observed data highlighted in white:

glm$post.dist


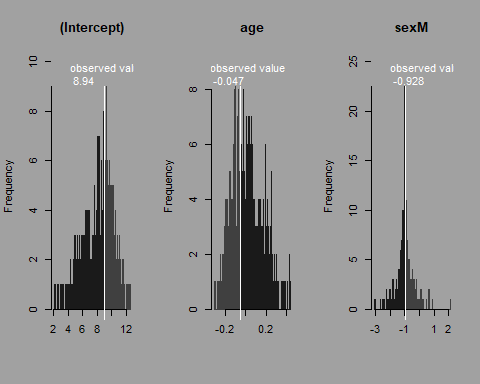


## 6.3 Multiple networks analysis

### 6.3.1 GLMM ANTs Function

GLMM test is used when the data contain ‘random factors’ (*e.g* for temporal and/or multiple groups study).
We will test for differences between degrees according to age and sex while controlling for groups with ANTs *stat.glmm* function.

*stat.glmm* is based on *R pacakge* [**lme4**](https://cran.r-project.org/web/packages/lme4/index.html) functions lmer,glmer and glm.nb and thus handles the same arguments.
*stat.glmm* has two extra arguments:

- 1. *ant* for the permuted data
  2. *progress* to print the progression of the analysis

### 6.3.2 Considerations when running GLMM

The use of a permutation approach may lead some models to encounter convergence and/or optimization issues when performing some permutations. This can also originate from the complexity of the model specified in regards to the data set (e.g. too many fixed factors regarding the sample size). While running the first model (on the original data set), ANTs checks for errors and warnings and asks if you want to continue. While computing the model on the permuted data, each time ANTs finds a warning or an error, a new permutation is done until the warning and error message disappears. Two considerations are required at that point:

1. The function can get stuck in an infinite loop when the model always returns error and/or warning messages.
2. By repeating the permutation process on the data each time an error and/or a warning is found, the posterior distribution will be forcibly restrained in a specific range of values. This is why the *stat.glmm* function will return the type of error or warning found while computing the models and the id of the permuted data set that caused the error and/or warning. This way, you have four options:
   1. Find the origin of the errors or warnings, create a new simpler model, scale and/or center variables, check singularity, or many other possibilities as explained in [Ben Bolker](https://bbolker.github.io/mixedmodels-misc/glmmFAQ.html#troubleshooting)’s GitHub web page.
   2. Compute diagnostic tests on the model with *ant* function.
   3. Erase the corresponding values of the posterior distribution that have been computed.
   4. Use the option control = [g]lmerControl(calc.derivs = FALSE) that turns off the derivative calculation performed after optimization. By doing so, most of the warnings can be discarded as explained in lme4 vignette: [lme4 Performance Tips](https://cran.r-project.org/web/packages/lme4/vignettes/lmerperf.html).

As such concern has not been addressed in the literature, it is up to the user to choose any of the options currently available.

### 6.3.3 Running GLMM

GLMM = stat.glmm(ant = pld,formula = degree ~ sex + age + (1|group), family = 'gaussian', progress = TRUE)

GLMM$errors

## NULL

Similar analyses can be done with generalized linear model with family types other than gaussian.
For GLMM, the *ant* function returns an object of four elements:

1. A summary of the original GLMM model with the data frame of statistics of permutation test:

glmm = ant(GLMM)


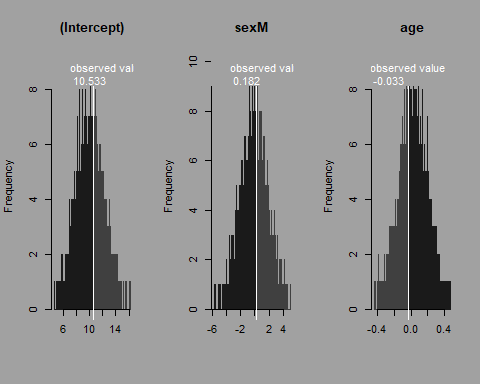


glmm$model

## Linear mixed model fit by REML ['lmerMod']
## Family: gaussian
## Formula: degree ~ sex + age + (1 | group)
## Data: odf
##
## REML criterion at convergence: 182.9
##
## Scaled residuals:
## Min 1Q Median 3Q Max
## -2.2768 -0.5423 0.1406 0.4653 1.5704
##
## Random effects:
## Groups Name Variance Std.Dev.
## group (Intercept) 0.00 0.00
## Residual 17.23 4.15
## Number of obs: 33, groups: group, 2
##
## Fixed effects:
## Estimate Std. Error t value p.left p.rigth p.one.side
## (Intercept) 10.53250 1.83046 5.75402 0.11781 0.88219 0.23562
## sexM 0.18170 1.46366 0.12414 0.89069 0.10931 0.21862
## age -0.03310 0.13815 -0.23962 0.78138 0.21862 0.43724
## lower.ci uper.ci mean
## (Intercept) 9.97599 10.03897 10.007
## sexM -0.12141 -0.06440 -0.093
## age 0.02225 0.02733 0.025
##
## Correlation of Fixed Effects:
## (Intr) sexM
## sexM -0.381
## age -0.854 0.050

1. Two plots of diagnostic of the original model:

glmm$model.diagnostic


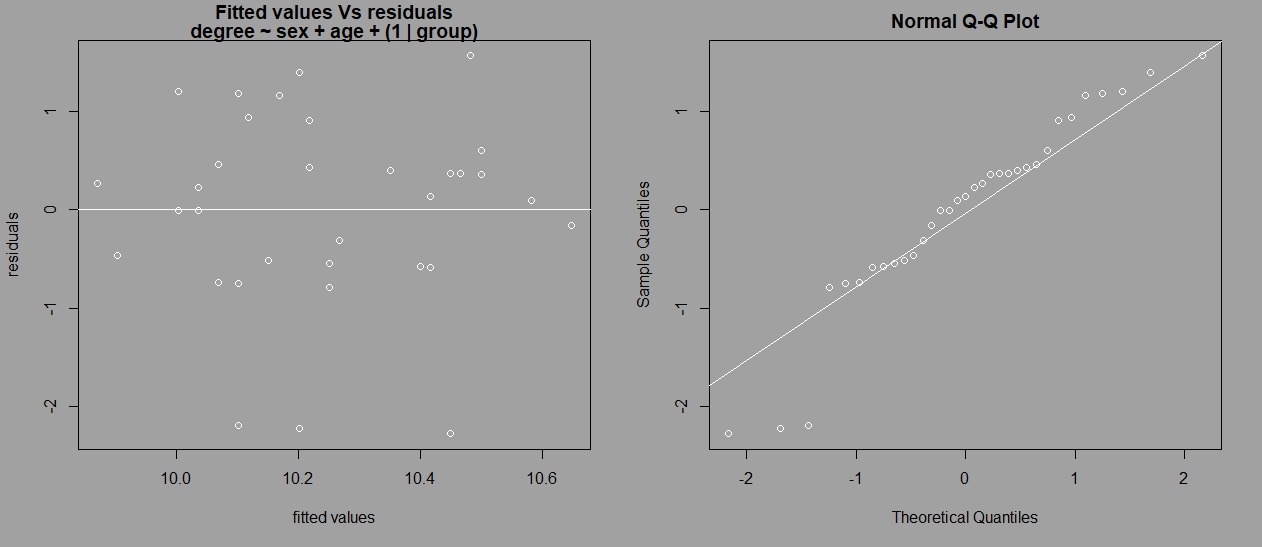


1. A histogram of the post-distribution of the statistics of interest with the value of the statistics of interest for the observed data highlighted in white:

glmm$post.dist


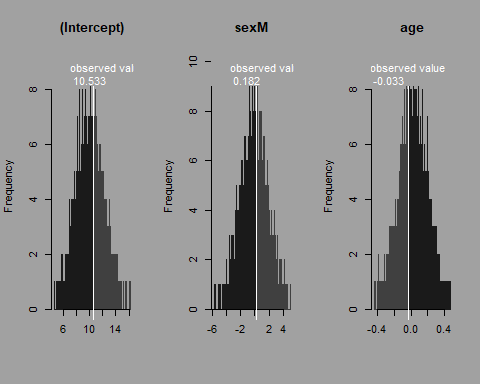


1. A vector of indices indicating the permutations with warnings or errors (these permutations have been re-permuted internally and the current values of the test correspond to the re-permuted data):

# Summary

In this manuscript we show how ANTs allows to easily run social network analysis on raw data by computing network metrics, permuting node labels and run permuted statistical test. The different steps can be synthetized as follows:

1. Import a data frame of interactions with ***read.csv(...)****.*
2. Convert this data frame into an adjacency matrix of interactions with ***df.to.mat(...)****.*
3. Import a data frame of individual characteristics with ***read.csv(...)****.*
4. Compute a node network metric with the functions of family ***met*.**.
5. Perform node label permutations with *perm.net.nl* ***(...)****.*
6. Run permuted statistical tests with the functions of type ***stat.***.
7. Compute statistics diagnostic of the analysis with permutation tests with ***ant()*** function.

All these steps have been tailored in generic functions allowing the user to have an all-in-one toolbox, similar to UCINET ([Borgatti, Everett & Freeman 2002](#_ENREF_1)) and SOCPROG ([Whitehead 2009](#_ENREF_11)), but with the flexibility of the R environment. This allows non-expert users to follow specific analytical protocols, and expert users to use ANTs functions to speed up their specific analytical protocols.

# References

Borgatti, S.P., Everett, M.G. & Freeman, L.C. (2002) Ucinet for Windows: Software for social network analysis.

Borgeaud, C., Sosa, S., Sueur, C. & Bshary, R. (2017) The influence of demographic variation on social network stability in wild vervet monkeys. *Animal Behaviour,* **134,** 155-165.

Croft, D.P., Madden, J.R., Franks, D.W. & James, R. (2011) Hypothesis testing in animal social networks. *Trends in ecology & evolution,* **26,** 502-507.

Farine, D.R. & Whitehead, H. (2015) Constructing, conducting and interpreting animal social network analysis. *Journal of Animal Ecology,* **84,** 1144-1163.

Kawazoe, T. & Sosa, S. (2019) Social networks predict immigration success in wild Japanese macaques. *Primates,* **60,** 213-222.

Liao, Z., Sosa, S., Wu, C. & Zhang, P. (2018) The influence of age on wild rhesus macaques' affiliative social interactions. *American journal of primatology,* **80,** e22733.

Sosa, S. (2016) The influence of gender, age, matriline and hierarchical rank on individual social position, role and interactional patterns in Macaca sylvanus at ‘La Forêt des singes’: A multilevel social network approach. *Frontiers in psychology,* **7**.

Sosa, S. (2018) Social Network Analysis. *Encyclopedia of Animal Cognition and Behavior* (eds J. Vonk & T. Shackelford), pp. 1-18. Springer International Publishing, Cham.

Sosa, S., Pele, M., Debergue, E., Kuntz, C., Keller, B., Robic, F., Siegwalt-Baudin, F., Richer, C., Zhang, P. & Ramos, A. (2018a) Impact of group management and transfer on individual sociality in Highland cattle (Bos Taurus). *arXiv preprint arXiv:1805.11553*.

Sosa, S., Puga-Gonzalez, I., Hu Feng, H., Zhang, P., Xiaohua, X. & Sueur, C. (2018b) A multilevel statistical toolkit to study animal social networks: Animal Network Toolkit (ANT) R package. *bioRxiv*.

Whitehead, H. (2009) SOCPROG programs: analysing animal social structures. *Behavioral Ecology and Sociobiology,* **63,** 765-778.
