# Supplementary material for: A multilevel statistical toolkit to study animal social networks: the Animal Network Toolkit Software (ANTs) R package
Source: Sci Rep. 2020 Jul 27;10:12507. doi: 10.1038/s41598-020-69265-8 (PMC7385643; doi:10.1038/s41598-020-69265-8)
Supplement: Supplementary file 3 — Supplementary Information 3. [file 41598_2020_69265_MOESM3_ESM.docx]

A multilevel statistical toolkit to study animal social networks: the Animal Network Toolkit Software (ANTs) R package

Sebastian Sosa, Ivan Puga-Gonzalez, Fenghe Hu, Pansanel Jérôme, Xiaohua Xie, Cédric Sueur

**Examining node level in static or time-aggregated networks with R package ANTs through data stream permutations**

# Introduction

Animal Network Toolkit software (ANTs) is an R package designed to perform social network analysis of animal social systems. This package aims to provide users with tools that enable a correct use of their data and to facilitate the use of complex analytical approaches. Here, we provide a tutorial to perform social network analysis at the individual (node) level, in static or time-aggregated networks using a data stream permutation approach. This workflow is based on two different objects: adjacency matrices (representing individual associations) and data frames (containing individuals’ characteristics such as sex, age, hierarchical rank, etc.). Adjacency matrices are used to do the data stream permutations and calculate network node measures and the data frames to store calculated data and statistical tests. The tutorial is divided into four different sections: 1) performing data stream permutations according to the data collection protocol (group follow or focal sampling), 2) computing network measures, 3) replicating statistical tests on permutations, and 4) computing statistics on the posterior distribution of these permutation tests.

Data stream permutations were first used in ecology to answer questions regarding whether, in group living animals, indices of association among pairs of individuals were purely random or not (Bedjer et al. 1998; Whitehead 1999). Contrary to node label permutations, data stream permutations swap a single association in each permutation and this permutation can be controlled according to the time or location where the observation was made. This is particularly important in order to control for non-social factors affecting individuals’ associations. This feature is an important aspect of data stream permutations that generates better rates of false negatives (type II error) than when using network permutations (Farine 2017). However, unlike network permutations, data stream permutations do not allow to include directionality neither to change the number of alters of an individual, making it impossible to test some network measures such as the number of grooming behaviors received although this information may be of interest for specific questions related to grooming up to the hierarchy ([Tiddi, Aureli & Schino 2012](#_ENREF_9)) or varying numbers of social patterns after significant changes in the group ([Firth *et al.* 2017](#_ENREF_7)).

Data stream permutations require raw data in which individual associations are recorded according to several factors such as the day, the time or the location of the observations. ANTs currently handles two types of data stream permutations in accordance with the protocol applied for data collection: Group follow (Bedjer et al. 1998; Whitehead 1999) or focal sampling ([Farine 2017](#_ENREF_5)). Although the primary data structure varies depending on the data collection protocol, the rest of the analytical protocol remains the same once the permutation process is complete. All ANTs functions detect if the data input is a data frame (for single network protocols) or a list of data frames (for multiple networks protocol), so the same functions can apply to single or multiple networks. These are the different steps to run these two protocols from raw data to permuted statistical results: 1) Import data of associations, 2) Perform data stream permutations 3) Import (a) data frame(s) of individual characteristics, 4) Compute node measures, 5) Permute statistical tests and results. If you have already read ANTs’ tutorial “Examining node level in static or time-aggregated networks with R package ANTs through node label permutations”, you should be familiar with parts 1, 3,

4 and 5.

In Appendix 4 you can download a zip file in which data for simulated single networks and multiple networks can be found.

# 1. Install ANTs

To date, ANTs is not available in CRAN but only in GitHub and must be installed via the R package *devtools* with the following command:

devtools::install_github('SebastianSosa/ANTs')

# 2. Import (a) data frame(s) of associations

library(ANTs)

## 2.1. Group follow protocol

Group follow protocol is a specific way to collect individual associations, often used by ecologists to collect data on populations. It consists in observing individual associations according to several spatial locations. Those different spatial locations represent the scans (*e.g.* individuals associations per spatial locations) (Altmann [1974](#ref-altmann1974observational)). Of course, when collecting data, other factors can be considered such as the day, the time of the day, etc. The combination of one or several control factors generates a new category of scans (*e.g.* individuals associations per spatial locations per days of observations). A permutation for such data collection protocol is made between two individuals from different observations with the condition that they were not observed together in the same observations.

ANTs data frame of associations for group follow protocol must comprise at least two columns:

1. A column indicating the ids of the individuals observed during the data collection. The column has to be named ‘ID’.
2. (A) column(s) indicating the type of scan(s) the user wants to use. The principle is to state which factor indicates an individual association (it can be a geographical location, a day, a time of the day or the combination of several factors).

A third column can indicate a *control factor* that constrains permutations within this control factor for the scan type stated, it can be a set of multiples columns of categorical or continuous factors.

An example of this type of data frame are given in the section [2.3.1 Group follow data type](##section1.3.1).

To import a data frame of interactions, use R function df=read.csv(...) once you have changed the R repertory in which you saved the file. Attached to this tutorial is a csv file named *associations.csv* with the simulated data.

## 2.2. Focal sampling protocol

Focal sampling protocol ([Altmann 1974](#_ENREF_1)) is widely used in primatology. It consists in following a specific individual during a certain amount of time and collecting individual behaviors. Recently, [Farine (2017)](#_ENREF_5) created a data stream approach for such data collection protocol showing its reliability. The principle is similar to other data stream approaches (*i.e.* permute two individuals from two different scans). In the focal sampling case, the scans are the different focals and the permutation is made between the alters (*i.e.* individuals with whom the focal interacts) of the two focal individuals selected with the condition that the alters permuted are not present in each other’s focal.

In ANTs, a data frame of interactions must comprise at least three columns:

1. A column indicating the ids of the focal individuals.
2. A column indicating the ids of the individuals with whom the focal individual interacts (one per line).
3. A column that distinguishes each focal (usually there are several focals per individual).

An example of this type of data frame are given in the section [2.3.2 focal sampling data type](#section1.3.2).

## 2.3. Importing a single data frame of associations

To import a single data frame of associations, use R function *df =read.csv(...)* once you have changed the R working directory in which you saved the file.

### 2.3.1 Group follow data type

In Appendix 4 in the following directory you can find the simulated associations for a single network: ‘single network/pre-network/group follow/ associations.csv’. Set your working directory and import the data.

setwd("Your.Path.../tutoriels/single network/data stream/group follow")
dg = read.csv(file = 'associations.csv', header = TRUE)

Example of data frame of group follow associations:

head(dg)

| day | time | location | ID | type_interaction |
| --- | --- | --- | --- | --- |
| 2 | morning | 3 | A | 2 |
| 1 | morning | 4 | B | 2 |
| 3 | morning | 1 | C | 1 |
| 3 | afternoon | 3 | F | 2 |
| 3 | morning | 1 | C | 1 |
| 2 | afternoon | 1 | D | 1 |
| 3 | afternoon | 4 | I | 1 |
| 1 | afternoon | 4 | C | 2 |
| 2 | afternoon | 3 | B | 1 |
| 3 | afternoon | 4 | G | 2 |
| 1 | morning | 1 | G | 2 |
| 1 | afternoon | 2 | J | 2 |
| 3 | morning | 2 | F | 1 |

These data simulate observations of several individuals (column 4: ‘ID’) on different days (column 1: ‘day’), at different times of the day (column 2: ‘time’), at different geographical locations (column 3: ‘location’) and for different types of behaviors (column 5: ‘type_interaction’). Individuals with the exact same values for each of the factors (day, time and location) are considered to belong to the same scan.

The choice of the appropriate scan is left to the user. For example, you can decide to use only one of these factors to build your scan category, like the day, so the permutations will be done between individuals observed on different days. But you can also create a combination of different factors to create a scan category, like the combination of day and time, so the permutations will be done between individuals observed on different days and at different times of the day. Finally, you can use a control factor to force permutations to happen according to a third factor. This factor can also be a combination of several factors contained in different columns. For example, assuming that the scan category was established according to the day, you can ‘force’ permutations between individuals observed on different days according to the location where the individuals were observed by declaring the column ‘location’ as control factor. This way, permutations will be done between individuals observed on different days but only if they were observed in the same location.

By allowing the creation of scan and control factor categories by combining several factors, ANTs provides a powerful and flexible approach to let the user create his own appropriate Null Hypothesis Test (NHT) (Farine [2017](#ref-farine2017guide)) according to the hypothesis of the research.

### 2.3.3 Convert Socprog data frame

Socprog ([Whitehead 2009](#_ENREF_10)) is a well-known social networking software. For those familiar with it, the previous section shows a data frame structure in a ‘linear’ mode in socprog. For those used to organize their data in a ‘dyadic’ or ‘group’ socprog data format, you can use the follow function to convert them into a linear mode: *convert.socprog*.

*convert.socprog* function has the following arguments:

1. *df* a data frame socprog style.
2. *id* an integer or string indicating the column of ids.
3. *scan* an integer or string vector indicating the column of the scan(s).
4. *sep* a character indicating the type of separation between individuals inside the column id.

#simulating group socprog format
scan = c(1:5)
id = c("A;E;T", "R;Y;B", "T;W;Y", "A;B;U"," A;B;Y")
location= c('a1', 'b1', 'a1', 'a1', 'b1')
D = data.frame(scan, location, id)
#Converting data frame
head(convert.socprog(D, id = 3, scan = c(1,2), sep = ';'))

| ## | scan | id |
| --- | --- | --- |
| ## | 1_a1 | A |
| ## | 1_a1 | E |
| ## | 1_a1 | T |
| ## | 2_b1 | R |
| ## | 2_b1 | Y |
| ## | 2_b1 | B |

### 2.3.2 Focal sampling data type

In Appendix 3 in the following directory you can find the simulated associations for a single network: ‘single network/pre-network/ focal sampling/group follow/ focal.csv’ Set your working directory and import the data.

setwd("Your.Path.../tutoriels/single network/data stream/focal sampling')
df = read.csv(file = 'focal.csv', header = TRUE)

Example of data frame of focal sampling associations:

head(df)

| # | nfocal | time |  | focal |  | alter |
| --- | --- | --- | --- | --- | --- | --- |
| # | 1 |  | afternoon | L |  | H |
| # | 1 |  | morning | F |  | N |
| # | 1 |  | afternoon | O |  | N |
| # | 1 |  | morning | F |  | H |
| # | 1 |  | afternoon | G |  | N |
| # | 1 |  | afternoon | B |  | I |

## 2.4. Importing multiple data frames of associations

When studying a group over several periods and/or when studying several groups (of the same species or not) you will obtain several independent networks in which data stream permutations have to be run separately. In order to analyze these data frames while controlling for independence, ANTs needs to store the data frames in a list format where each element represents a group and/or a period of observations. For example, if you have two groups observed over two periods, you will have 4 data frames (one for each period for each group), like in the simulated data that we will use. Each of the data frames has to be structured in the same way, explained in section [1. Import a data frame(s) of associations](file:///C:\Users\Sebastian\Documents\ANT%20unofficial\tutoriels\section1), *i.e.*that all the columns must be arranged in the same order. To analyze this type of data, ANTs allows you to run permuted Generalized Linear Mixed Models in order to control for these ‘segregational’ factors.

In this tutorial, we simulated the associations of two groups of individuals that are characterized by their ids and also by their age and sex. To import several data frames of associations at once, use the ANTs function import.df once you have changed the R working directory in which you saved all the data frames in separate ‘.csv’ files without any other file. import.df function imports all the files present in the R working directory in the form of a list, each element of the list named according to the corresponding ‘.csv’ file and ordered alphabetically. *import.df* function has the same arguments as R function read.csv and thus can handle different separators, coma formats, files with headers and row names, etc. (for more information, type *?read.csv* or *?import.df*).

### 2.4.1 Group follow multiple networks

In Appendix 3 in the following directory you can find the simulated associations for multiple networks: ‘multiples networks/pre-network/group follow/associations’ with two csv files ‘group1.csv’ and ‘group2.csv’. Select your working directory and import your data frames of associations of group follow protocol:

setwd("Your.Path.../tutoriels/multiples groups/Data stream/group follow/associations")
ldg = import.df()

Example of data frames of group follow associations:

head(ldg)

### 2.4.1 Focal sampling multiple networks

In Appendix 3, in the following directory you can find the simulated associations for multiple networks: ‘multiple networks/ pre-network/focal sampling/group follow/observations’ with two csv files ‘group1.csv’ and ‘group2.csv’. Select working directory and import your data frames of associations of focal sampling protocol:

setwd("Your.Path.../tutoriels/multiples groups/data stream/focal sampling/observations")
ldf = import.df()

Example of data frames of focal sampling associations:

ldf

# 3. Import (a) data frame(s) of individual characteristics

As explained in the abstract, ANTs works in synergy between adjacency matrix(ces) (representing individual associations) and (a) data frame(s) in a supplementary file (representing individual characteristics such as sex, age, hierarchical rank, etc.). The adjacency matrix(ces) are used to compute common network node measures and the data frame stores these network node measures, on which the data stream permutations will be done and statistical tests will be run.

## 3.1. Importing a single data frame of individual characteristics

Like for importing a data frame of associations, use the R function *df=read.csv(...)* once you have changed the R repertory in which you saved the file. Attached to this tutorial is a csv file named ‘attr.csv’ with the simulated data.

In Appendix 3, in the following directory you can find the simulated individual characteristics: ‘single network/pre-network/ focal sampling/group follow/ characteristics.csv’. Set your working directory and import the data.

setwd("Your.Path.../tutoriels/single network/data stream/group follow')

Import your data frame of individual characteristics:

dig = read.csv(file = 'characteristics.csv', header = TRUE)

Example of data frame of individual characteristics:

head(dig)

Repeat the same process for individual characteristics for focal sampling data inside the ‘single network/pre-network/ focal sampling’ directory:

setwd("Your.Path.../tutoriels/single network/data stream/focal sampling")
dif = read.csv(file = 'characteristics.csv', header = TRUE)

If you do not have a data frame of individual characteristics, ANTs allows you to create an empty data frame with the same size as the matrix with the function *df.create(...).*

## 3.2. Importing multiple data frames of individual characteristics

As for importing multiple data frames of individual associations, use the ANTs function import.df to import multiple data frames of individual characteristics.

These files must be in a separate folder from those of the data frames (or matrices) of associations and must be named identically as the import.df function imports the files following an alphabetical order.

Each data frame must hold (a) column(s) indicating the factor(s) that identify each network and is/are considered random factors in the GLMM you will use *i.e* add (a) column(s) indicating the value of the random factor(s) in each data frame (in your case, the column group). Attached to this tutorial is a file named ‘multiple groups individual characteristics’ with the simulated data.

Inside the ‘multiple networks/pre-network/group follow/characteristics’ directory, you will see two csv files called ‘group1.csv’ and ‘group2.csv’. Change your directory and import your data frames of individual characteristics for group follow protocol:

setwd("Your.Path.../tutoriels/multiples groups/data stream/group follow/characteristics")
ldig = import.df()

Do the same for data frames of individual characteristics for focal sampling protocol once you have changed your work directory to ‘multiple networks/pre-network/focal sampling/characteristics’:

setwd("Your.Path.../tutoriels/mmultiples groups/data stream/group follow/characteristics")
ldif = import.df()

If you do not have a data frame of characteristics, ANTs allows you to create an empty data frame with the same size as the matrix with the function *df.create(...)*.

# 4. Perform data stream permutations

Data streamstream permutations is a useful approach when the data collected concern individuals associations, in which the level of uncertainty is high ([Croft *et al.* 2011](#_ENREF_4)).Here, the null hypothesis is to test if nodes’ measures are randomly distributed according to individual associations.
To perform data stream permutations with ANTs, use the function of type perm.ds.. There are currently two types of perm.ds. available in ANTs:

1. *perm.ds.group* for data originating from group follow data collection protocol.
2. *perm.ds.focal* for data originating from focal sampling data collection protocol.

## 4.1 Data stream permutations for group follow

The perm.ds.group function contains six arguments:

1. *df* is a data frame of associations described in section [1.1. Group follow protocol](##section1.1) .
2. *scan* is an integer or a string indicating the column of scans of individual associations.
3. *control_factor* is an optional integer or a string indicating the column(s) of confounding factor(s) by which to control scan associations. The types of control factors can be continuous or categorical.
4. *index* is a string indicating the type of association index used to create the association matrices. There are currently three association indexes available in ANTs:

a. ‘sri’ for Simple ratio index:

$$\frac{x}{x}+yAB+yA+yB$$

b. ‘hw’ for Half-weight index:

$$x/(x+yAB+1/2(yA+yB))$$

c. ‘sr’ for Square root index:

$$x/sqr((x+yAB+yA)(x+yAB+yB))$$

1. *nperm* is an integer that indicates the number of permutations to perform.
2. *progress* is an optional boolean that indicates the progression of the permutation process. By default, this option is equal to TRUE. However, this option may slow down the permutations.

This function returns a list of association matrices of length *nperm+1* with the first element being the original association matrix and the others are permuted association matrices.

Pdg = perm.ds.grp(df = dg, scan = ' location', nperm = 1000, progress = TRUE, index ='sri')

## 4.2 Data stream permutations for focal sampling

The perm.ds.focal function contains six arguments:

1. *df* is a data frame of associations described in section [1.2. focal sampling protocol](#section1.2).

2. *focal* is an integer or a string indicating the column of the focal individual.

3. *ctrl* is an integer or a string indicating the column(s) of the ids of focal individuals.

4. *alters* is an integer or a string indicating the column of alters.

5. *nperm* is an integer indicating the number of permutations to perform.

6. *progress* is an optional boolean that indicates the progression of the permutation process. By default, this option is equal to TRUE. However, this option may slow down the permutations.

This function returns a list of association matrices of length *nperm+1* with the first element being the original association matrix and the others are permuted association matrices.

Pdf = perm.ds.focal(df, focal = 'focal', ctrl = c(1,3), alters = 'alter', nperm = 1000, progress = TRUE)

## 4.3. Data stream permutations for multiple networks

For a multiple analysis approach, simply run the corresponding function on the list of association data you import through the function import.df in section [2.2 Importing multiple data frames of associations](#section2.2).

### 4.3.4 Multiple groups follow

pldg = perm.ds.grp(df = ldg, scan = 'location', perm = 1000, progress = TRUE, index ='sri')

### 4.3.5 Multiple focals

pldf = perm.ds.focal(ldf, focal = 'focal', ctrl = c(1,3), alters = 'alter', nperm = 1000, progress = TRUE)

# 5. Compute node measures

Now you have the adjacency matrix and the data frame. ANTs allows you to compute several node measures (for an overview of them see [Sosa (2018)](#_ENREF_8)). All of them belong to the type met.:

1. Degree (*met.degree*)
2. Indegree (*met.indegree*)
3. Outdegree (*met.outdegree*)
4. Strength (*met.strength*)
5. Instrength (*met.instrength*)
6. Outstrength (*met.outstrength*)
7. Eigenvector (*met.eigenvector*)
8. Laplacian centrality (*met.lp*)
9. Reach (*met.reach*)
10. Disparity (*met.disparity*)
11. Affinity (*met.affinity*)
12. R-I index (*met.ri*)
13. Betweenness (*met.betweenness*)

ANTs graphical user interface allows you to compute all these node measures using the function *met*.

## 5.1. Possible outputs

ANTs’ *met* functions take a matrix as main argument. The functions detect if this input is a single matrix (for a single network protocol) or a list of matrices (for a multiple network protocol). Thus, the same functions can be used to compute any of the network measures present in ANTs. The output of the *met* functions can be either a vector with the values of each node measure:

met.strength(pdf[[1]])

## H N I O G J Q
## 1.7395604 0.8322796 1.1080087 0.7573676 0.7925016 0.4967033 0.0000000
## D C M E A B K
## 0.7871795 0.6345238 0.9199134 0.6040404 0.7857143 1.0475957 2.2125763
## L F Z Y
## 1.1913420 1.0136364 0.0000000 0.3333333

or a data frame, in which one of the columns will contain the node measure calculated by the *met* function. For the data frame output, the argument *df* of the *met* function should be a data frame with at least one column containing individuals’ IDs. Note that rows’ and columns’ names of the input matrix have to be in the same order as the IDs’ column of the input data frame (this may be the case when you start with an edge list of interactions because the matrix generated is arranged according order of apparition of individuals in the edge list of interaction). To order the data frame according to the matrix(ces) you can use the argument *dfid* of the *met* function to indicate the IDs’ column of the input data frame. In this way, ANTs will match the IDs of the input data frame to the IDs of the input matrix and create a column in the output data frame with the node measure in that specific ID order. Note that to perform permuted statistical tests, node measures must be stored in the data frame.

head(met.strength(pdf[[1]], df =dif, dfid = 1))

| # | id | sex | age | strength |
| --- | --- | --- | --- | --- |
| # | 8 | H | male | 6 1.7395604 |
| # | 14 | N | male | 3 0.8322796 |
| # | 9 | I | male | 3 1.1080087 |

Now, we will store this node measure in the data frame of individual characteristics to perform node label permutations by declaring:

- 1. *df*, the argument specifying the data frame in which to perform data stream permutations
  2. *dfid* and optional argument, to declare which column of the data frame stores the individuals’ IDs, which will help ANTs function to merge the node measure with the corresponding individuals in the data frame.

dig = met.strength(pdg, df = dig, dfid=1)

We will do the same for the list of data frames.

Ldig = met.strength(pldg, df = ldig, dfid = 1)

As you can see, the data frames have been merged per random factors and now your two groups are merged and ready for GLMM analysis.

Head(ldig[[1]])

| # | id | sex | age | group | strength |
| --- | --- | --- | --- | --- | --- |
| # | A | male | 7 | group1 | 14.8 |
| # | B | female | 3 | group1 | 14.8 |
| # | C | female | 7 | group1 | 14.8 |
| # | D | male | 11 | group1 | 14.8 |
| # | E | male | 8 | group1 | 10.2 |
| # | F | male | 7 | group1 | 14.8 |
| # | G | male | 6 | group1 | 12.3 |
| # | H | female | 2 | group1 | 14.8 |
| # | I | male | 5 | group1 | 7.033333 |
| # | J | female | 5 | group1 | 10.33333 |
| # | a | female | 9 | group2 | 28.2 |
| # | b | female | 4 | group2 | 13.8 |
| # | c | male | 5 | group2 | 22.8 |
| # | d | female | 9 | group2 | 15.06667 |

# 6. Run permuted statistical tests & compute permuted statistical results

ANTs allows to run the following statistical tests on permuted data using the functions of type *stat.*:

1. Correlation
2. T-test
3. LM/GLM
4. GLMM
5. Assortativity testTauKr test
6. Deletion simulations

In this tutorial, we will only see how to perform permuted [correlation tests](file:///C:\Users\Sebastian\Dropbox\Redactions\Papers%20&amp;%20books\ANT\3.%20Node%20label%20permutations\BiorXiv\Examining%20node%20level%20in%20static%20or%20time-aggregated%20networks%20with%20R%20package%20ANTs.docx#section5.3.1), [t-tests](file:///C:\Users\Sebastian\Dropbox\Redactions\Papers%20&amp;%20books\ANT\3.%20Node%20label%20permutations\BiorXiv\Examining%20node%20level%20in%20static%20or%20time-aggregated%20networks%20with%20R%20package%20ANTs.docx#section5.3.2) and [LM test](file:///C:\Users\Sebastian\Dropbox\Redactions\Papers%20&amp;%20books\ANT\3.%20Node%20label%20permutations\BiorXiv\Examining%20node%20level%20in%20static%20or%20time-aggregated%20networks%20with%20R%20package%20ANTs.docx#section5.3.3), [GLM test](file:///C:\Users\Sebastian\Dropbox\Redactions\Papers%20&amp;%20books\ANT\3.%20Node%20label%20permutations\BiorXiv\Examining%20node%20level%20in%20static%20or%20time-aggregated%20networks%20with%20R%20package%20ANTs.docx#section5.3.4) and [Generalized Linear Mixed Models](file:///C:\Users\Sebastian\Dropbox\Redactions\Papers%20&amp;%20books\ANT\3.%20Node%20label%20permutations\BiorXiv\Examining%20node%20level%20in%20static%20or%20time-aggregated%20networks%20with%20R%20package%20ANTs.docx#section5.4) (GLMM) as we are currently working at a node level.

## 6.2. ANTs statistical results

ANTs allows to run a diagnostic test from permuted statistics obtained through the functions of type *stat*. with the method *ant*. This method adapts the diagnostic results according to the data input. The output is adapted to the type of test run. However, some outputs are common to all tests, a data frame of statistics of permutation tests:

1. P-values, by the nature of the test, they are one-tailed p-values, on the right and left of the distribution.
2. Measurement of the effect size of the post-distribution according to the statistics of interest ([Farine & Whitehead 2015](#_ENREF_6)), 95% confidence interval, and mean.
3. A histogram of the post-distribution of the statistics of interest with the value of the statistics of interest for the real data highlighted in white.

## 6.3 Single network analysis

### 6.3.1 Correlation

We will start with a simple correlation between age and degree with the function *stat.cor*.
Based on R *cor* function, *stat.cor* handles the same arguments, consequently allowing Pearson, Spearman and Kendall correlations. *stat.cor* has two extra arguments:

1. *ant* for the permuted data
2. progress to print the progression of the analysis

C = stat.cor(ant = dig, 'age', 'strength', method = 'pearson', progress = TRUE)
c = ant(C)

The *ant* function returns a list of two elements:

For correlation test, the *ant* function returns an object with:

1. A summary of the statistical test with the data frame of statistics of permutation test:

c$statistics

## Observed correlation p.left p.right p.one.side 95ci lower
## statistics -0.06882411 0.788 0.204 0.408 -0.2123282
## 95ci upper mean
## statistics -0.1976233 -0.2049757

1. A histogram of the post-distribution of the statistics of interest with the value of the statistics of interest for the observed data highlighted in white:

c$post.dist


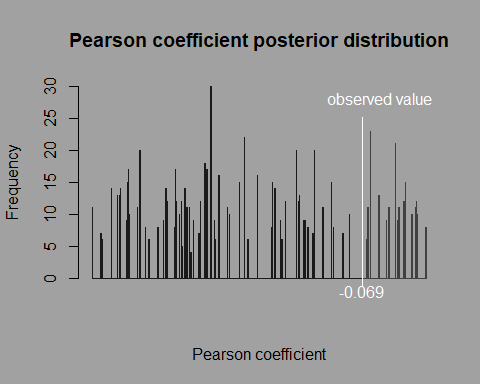


### 6.3.2 T-test

We will continue with a t-test to test for sex differences in the measure degree with the function *stat.t.* This function is based on the R function *t.test*.

*stat.t* handles the same arguments as in the *s3* formula method, with two extra arguments:

1. *ant* for the permuted data
2. *progress* to print the progression of the analysis

TT = stat.t(ant = dig, formula = strength ~ sex, progress = FALSE)
t = ant(TT)

For T-test, the *ant* function returns an object of two elements:

1. A summary of the statistical test with the data frame of statistics of permutation test:

## t observed p.left p.right p.one.side 95ci lower 95ci upper
## statistics 0.008218284 0.186 0.754 0.372 0.1744832 0.2190349
## mean
## statistics 0.196759

1. A histogram of the post-distribution of the statistics of interest with the value of the statistics of interest for the observed data highlighted in white:

t$post.dist


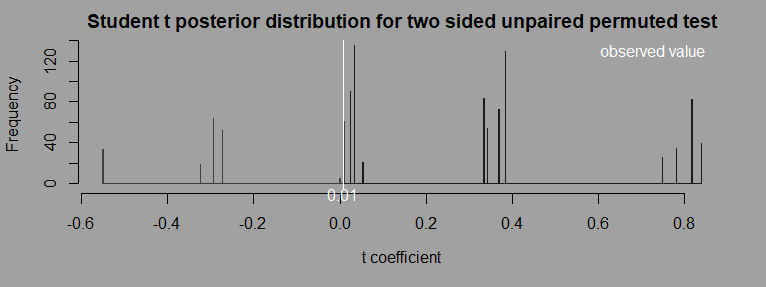


### 6.3.3 LM

Now we will run a linear model to simultaneously test for age and sex differences with the function *stat.lm*.

Based on R *lm* function, *stat.lm* handles the same arguments with 2 extra arguments:

1. *ant* for the permuted data
2. *progress* to print the progression of the analysis

LM = stat.lm(ant = pd, formula = strength ~ age + sex, progress = TRUE)
l = ant(LM)

For linear models, the *ant* function returns an object with three elements:

1. A summary of the original LM model with the data frame of statistics of permutation test:

l$model

##
## Call:
## "strength ~ age + sex"
##
## Residuals:
## Min 1Q Median 3Q Max
## -3.3125 -0.6891 0.0020 1.5548 1.7478
##
## Coefficients:
## Estimate Std. Error t value p.left p.rigth p.one.side
## (Intercept) 13.88930 1.94948 7.12461 0.19800 0.79400 0.39600
## age -0.03122 0.15563 -0.20062 0.83000 0.16200 0.32400
## sexmale 0.12280 1.47294 0.08337 0.53700 0.45500 0.91000
## lower.ci uper.ci mean
## (Intercept) 14.54162 14.61691 14.579
## age -0.08857 -0.08325 -0.086
## sexmale 0.15807 0.19951 0.179
##
## Residual standard error: 1.909 on 7 degrees of freedom
## Multiple R-squared: 0.005724, Adjusted R-squared: -0.2784
## F-statistic: 0.02015 on 2 and 7 DF, p-value: 0.9801

1. Two plots of diagnostic of the original model :

l$model.diagnostic


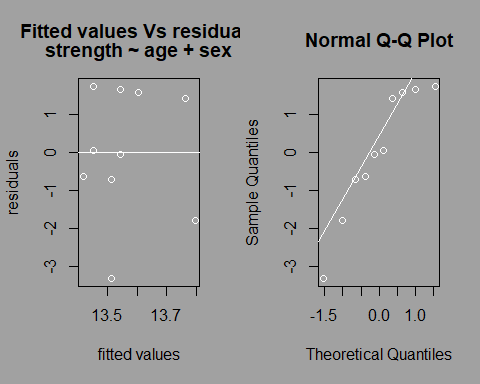


1. A histogram of the post-distribution of the statistics of interest with the value of the statistics of interest for the real data highlighted in white:

l$post.dist


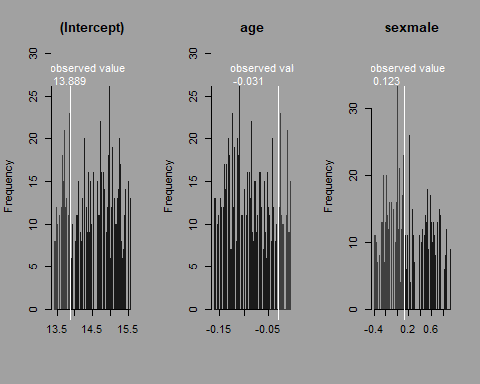


### 6.3.4 GLM

Similar analyses can be done with generalized linear models with different types of family distribution.
Based on R *glm* function, *stat.glm* handles the same arguments with 2 extra arguments:

1. *ant* for the permuted data
2. *progress* to print the progression of the analysis

GLM = stat.glm(ant = dig,formula = strength ~ age + sex, progress = TRUE, family = 'gaussian')
GLM = ant(GLM)

For GLM, the *ant* function returns an object of three elements:

1. A summary of the original GLM model with the data frame of statistics of permutation test:

GLM$model

##
## Call:
## "strength ~ age + sex , family = gaussian"
##
## Deviance Residuals:
## Min 1Q Median 3Q Max
## -3.3125 -0.6891 0.0020 1.5548 1.7478
##
## Coefficients:
## Estimate Std. Error t value p.left p.rigth p.one.side
## (Intercept) 13.88930 1.94948 7.12461 0.19800 0.79400 0.39600
## age -0.03122 0.15563 -0.20062 0.83000 0.16200 0.32400
## sexmale 0.12280 1.47294 0.08337 0.53700 0.45500 0.91000
## lower.ci uper.ci mean
## (Intercept) 14.54162 14.61691 14.579
## age -0.08857 -0.08325 -0.086
## sexmale 0.15807 0.19951 0.179
##
## (Dispersion parameter for gaussian family taken to be 3.642459)
##
## Null deviance: 25.644 on 9 degrees of freedom
## Residual deviance: 25.497 on 7 degrees of freedom
## AIC: 45.739
##
## Number of Fisher Scoring iterations: 2

1. Two plots of diagnostic of the original model:

GLM$model.diagnostic


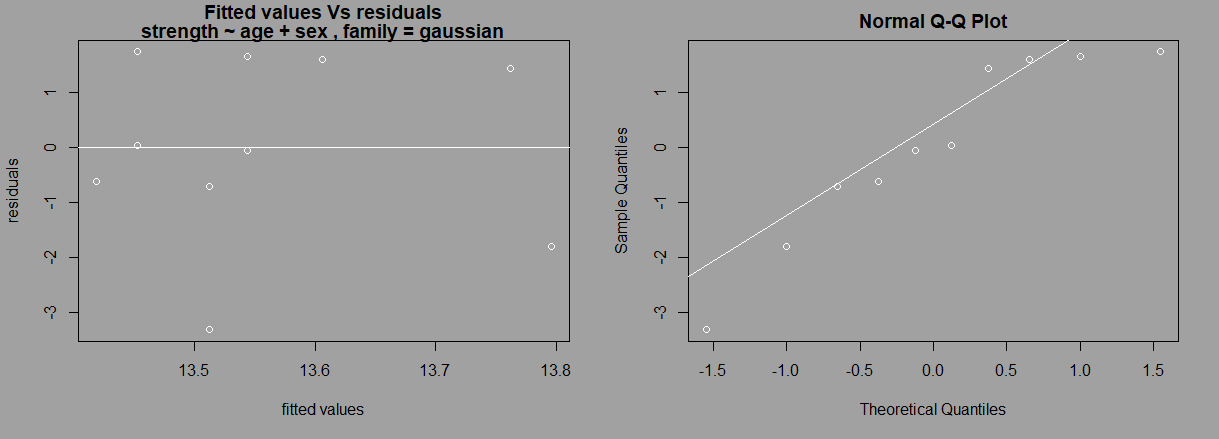


1. A histogram of the post-distribution of the statistics of interest with the value of the statistics of interest for the observed data highlighted in white:

GLM$post.dist


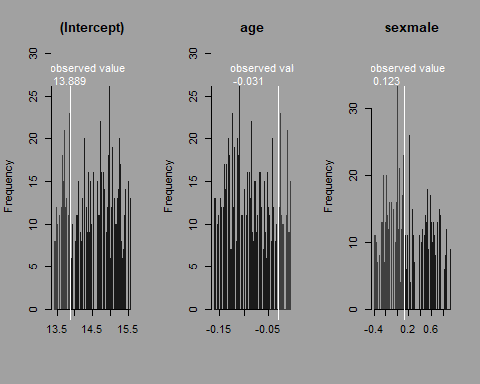


## 6.4 Multiple network analysis

### 6.4.1 GLMM ANTs function

GLMM test is used when the data contain ‘random factors’ (*e.g* for temporal and/or multiple groups study).
We will test for differences between degrees according to age and sex while controlling for groups with ANTs *stat.glmm* function.

*stat.glmm* is based on *R package* [**lme4**](https://cran.r-project.org/web/packages/lme4/index.html) ([Bates *et al.* 2014](#_ENREF_2)) functions lmer, glmer and glm.nb and thus handles the same arguments.

*stat.glmm* has two extra arguments:

- 1. *ant* for the permuted data
  2. *progress* to print the progression of the analysis

### 6.4.2 Considerations when running GLMM

The use of a permutation approach may lead some models to encounter convergence and/or optimization issues when performing some permutations. This can also originate from the complexity of the model specified in regards to the data set (e.g. too many fixed factors regarding the sample size). While running the first model (on the original data set), ANTs checks for errors and warnings and asks if you want to continue. While computing the model on the permuted data, each time ANTs finds a warning or an error, a new permutation is done until the warning and error message disappears. Two considerations are required at that point:

1. The function can be stuck in an infinite loop when the model always returns error and/or warning messages. Progress bar for progress visualization can help to detect such issue and the user can stop the process at any time.
2. By repeating the permutation process on the data each time an error and/or a warning is indicated, the posterior distribution will be forcibly restrained within a specific range of values. This is why the ‘*stat.lm*’, ‘*stat.glm*’ and ‘*stat.glmm*’ function will return the type of error or warning found while computing the models and the id of the permuted data set that caused the error and/or warning. The user has then four options:
   1. Find the origin of the errors or warnings, create a new simple model, scale and/or center variables, check singularity, or many other possibilities as explained in Ben Bolker github web page: <https://rstudio-pubs-static.s3.amazonaws.com/33653_57fc7b8e5d484c909b615d8633c01d51.html>
   2. Compute diagnostic tests on the model with ‘ant’ function.
   3. Use the option ‘*control = [g]lmerControl(calc.derivs = FALSE)*’ that turns off the derivative calculation performed after optimization. By doing so, most of the warnings can be discarded as explained in lme4 vignette:lme4 Performance Tips : <https://cran.r-project.org/web/packages/lme4/vignettes/lmerperf.html>

As such concerns have not been addressed in the literature, the choice of the most appropriate option is left to the user.

As such concern has not been addressed in the literature to date, it is up to the user to choose any of the options currently available.

### 6.4.3 Running GLMM

Similar analyses can be done with generalized linear models with different types of family distribution.

Based on R *glm* function, *stat.glm* handles the same arguments with 2 extra arguments:

1. *ant* for the permuted data
2. *progress* to print the progression of the analysis

GLMM = stat.glmm(ant = ldig, formula = strength ~ age + sex + (1|group), oda = ldg, family = 'gaussian', progress = TRUE)

Glmm = ant(GLMM)

For GLM, the *ant* function returns an object of three elements:

1. A summary of the original GLM model with the data frame of statistics of permutation test:

glmm$model

## Linear mixed model fit by REML ['lmerMod']
## Family: gaussian
## Formula: strength ~ age + sex + (1 | group)
## Data: odf
##
## REML criterion at convergence: 206.9
##
## Scaled residuals:
## Min 1Q Median 3Q Max
## -2.2944 -0.6360 0.2169 0.6492 1.5658
##
## Random effects:
## Groups Name Variance Std.Dev.
## group (Intercept) 37.92 6.158
## Residual 19.68 4.436
## Number of obs: 36, groups: group, 2
##
## Fixed effects:
## Estimate Std. Error t value p.left p.rigth p.one.side
## (Intercept) 16.71607 4.79973 3.48271 0.08600 0.91400 0.17200
## age 0.13194 0.24718 0.53378 0.90900 0.09100 0.18200
## sexmale -0.26376 1.49995 -0.17584 0.43600 0.56400 0.87200
## lower.ci uper.ci mean
## (Intercept) 17.72840 17.83994 17.784
## age -0.04844 -0.03198 -0.040
## sexmale -0.24724 -0.14614 -0.197
##
## Correlation of Fixed Effects:
## (Intr) age
## age -0.350
## sexmale -0.170 0.030

In data frame ‘Coefficients’ starting from p.left included, you can found the permuted statistics. Before that is the referential statistical results.

1. Two plots of diagnostic of the original model:

glmm$model.diagnostic


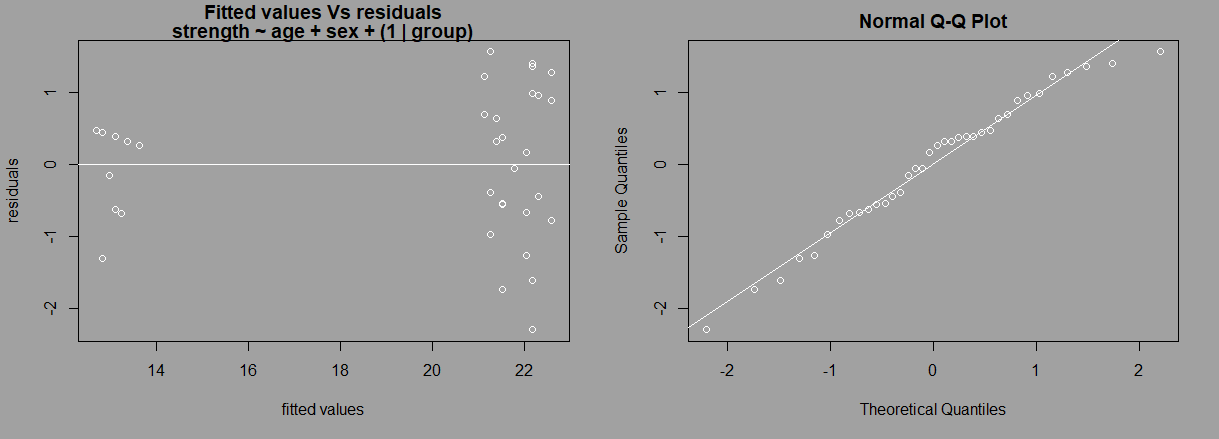


1. A histogram of the post-distribution of the statistics of interest with the value of the statistics of interest for the observed data highlighted in white:

glmm$post.dist


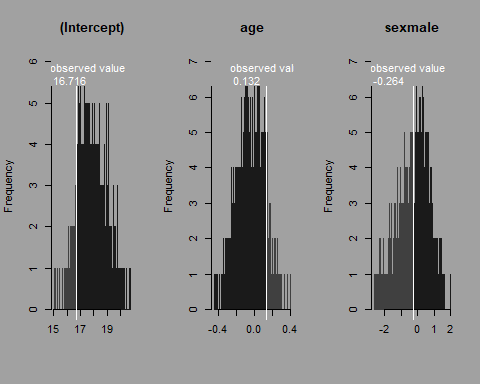


# Summary

In this tutorial we show how ANTs allows to easily run social network analysis on raw data by computing network measures, run data stream permutations and permuted statistical test. The different steps can be synthetized as follows:

1. Import a data frame of interactions with ***read.csv(...)***
2. Import a data frame of individual characteristics with ***read.csv(...)****.*
3. Perform data stream permutations with ***perm.ds.*** family functions.
4. Compute a node network metric with the functions of family ***met*.**.
5. Run permuted statistical tests with the functions of type ***stat.***.
6. Compute statistics diagnostic of the analysis with permutation tests with ***ant()*** function.

All these steps have been tailored in generic functions allowing the user to have an all-in-one toolbox, similar to UCINET ([Borgatti, Everett & Freeman 2002](#_ENREF_3)) and SOCPROG ([Whitehead 2009](#_ENREF_10)), but with the flexibility of the R environment. This allows non-expert users to follow specific analytical protocols, and expert users to use ANTs functions to fasten their specific analytical protocols.

# References

Altmann, J. (1974) Observational study of behavior: sampling methods. *Behaviour,* **49,** 227-266.

Bates, D., Mächler, M., Bolker, B. & Walker, S. (2014) Fitting linear mixed-effects models using lme4. *arXiv preprint arXiv:1406.5823*.

Borgatti, S.P., Everett, M.G. & Freeman, L.C. (2002) Ucinet for Windows: Software for social network analysis.

Croft, D.P., Madden, J.R., Franks, D.W. & James, R. (2011) Hypothesis testing in animal social networks. *Trends in ecology & evolution,* **26,** 502-507.

Farine, D.R. (2017) A guide to null models for animal social network analysis. *Methods in Ecology and Evolution*.

Farine, D.R. & Whitehead, H. (2015) Constructing, conducting and interpreting animal social network analysis. *Journal of Animal Ecology,* **84,** 1144-1163.

Firth, J.A., Voelkl, B., Crates, R.A., Aplin, L.M., Biro, D., Croft, D.P. & Sheldon, B.C. (2017) Wild birds respond to flockmate loss by increasing their social network associations to others. *Proceedings of the Royal Society B: Biological Sciences,* **284,** 20170299.

Sosa, S. (2018) Social Network Analysis. *Encyclopedia of Animal Cognition and Behavior* (eds J. Vonk & T. Shackelford), pp. 1-18. Springer International Publishing, Cham.

Tiddi, B., Aureli, F. & Schino, G. (2012) Grooming up the hierarchy: the exchange of grooming and rank-related benefits in a new world primate. *PloS one,* **7,** e36641.

Whitehead, H. (2009) SOCPROG programs: analysing animal social structures. *Behavioral Ecology and Sociobiology,* **63,** 765-778.
